# Supplementary material for: Food insecurity and the nutritional health and well-being of women and children in high-income countries: protocol for a qualitative systematic review
Source: BMJ Open. 2021 Aug 26;11(8):e048180. doi: 10.1136/bmjopen-2020-048180 (PMC8395272; doi:10.1136/bmjopen-2020-048180)
Supplement: Supplementary data [file bmjopen-2020-048180supp002.pdf]

Supplementary File B: List of high-income countries that meet the inclusion criteria (defined by list of OECD High-Income Economies). Available at: https://www.worldbank.org/en/news/press-release/2019/10/24/doing-business-2020-oecd-high-income-economies-remain-global-benchmarks-on-most-doing-business-indicators. Accessed November, 2020)

Australia
Austria
Belgium
Canada
Chile
Czech Republic
Denmark
Estonia
Finland
France
Germany
Greece
Hungary
Iceland
Ireland
Israel
Italy
Japan
Korea
Latvia
Lithuania
Luxembourg
Netherlands
New Zealand
Norway
Poland
Portugal
Slovakia
Slovenia
Spain
Sweden
Switzerland
United Kingdom
United States
