# Supplementary material for: Food insecurity and the nutritional health and well-being of women and children in high-income countries: protocol for a qualitative systematic review
Source: BMJ Open. 2021 Aug 26;11(8):e048180. doi: 10.1136/bmjopen-2020-048180 (PMC8395272; doi:10.1136/bmjopen-2020-048180)
Supplement: Supplementary data [file bmjopen-2020-048180supp003.pdf]

Supplementary File C - List of relevant stakeholder websites to be searched

- European Food banks Federation - https://www.eurofoodbank.org/
- Trussell Trust - https://www.trusselltrust.org/
- Independent Food Aid Network - https://www.foodaidnetwork.org.uk/
- The Food Foundation - https://foodfoundation.org.uk/
- Feeding Britain - https://feedingbritain.org/
- Feeding America - https://www.feedingamerica.org/
- Foodbanks Canada - https://www.foodbankscanada.ca/
- Foodbank Australia - https://www.foodbank.org.au/?state=au
- The Foodbank Project - https://www.foodbank.org.nz/
- Royal College of Gynaecology and Obstetrics - https://www.rcog.org.uk/
- Royal College of Nursing - https://www.rcn.org.uk/
- World Health Organiaation and affiliate websites (e.g., UNICEF, International Confederation of Midwives) - https://www.who.int/ https://www.unicef.org.uk/ https://www.internationalmidwives.org/
- Paediatrics; Nursing sub-specialties – Perinatal, Neonatal, Community, Family, Public Health; Midwifery, Dietitian and Doula coalitions and associations – websites from high resourced, Western countries (e.g., International Coalition of Nurses https://www.icn.ch/)
- Baby Friendly Initiative (formerly Baby Friendly Hospital Initiative) websites associated with high resourced, Western countries e.g. https://www.unicef.org.uk/babyfriendly/
- Breastfeeding and Lactation coalitions and associations – websites from high resourced, Western countries (e.g., La Leche League International https://www.llli.org/)
