# Supplementary material for: Food insecurity and the nutritional health and well-being of women and children in high-income countries: protocol for a qualitative systematic review
Source: BMJ Open. 2021 Aug 26;11(8):e048180. doi: 10.1136/bmjopen-2020-048180 (PMC8395272; doi:10.1136/bmjopen-2020-048180)
Supplement: Supplementary data [file bmjopen-2020-048180supp004.pdf]

Supplementary File D – Example search strategy

**Search Concept – Scopus**			**Rationale**
Population terms	S1	TITLE-ABS-KEY(wom?n OR female OR mother* OR matern* OR mum OR father OR dad* OR caregiver OR guardian OR parent* OR preg* OR postnatal OR newborn OR baby OR babies OR infan* OR toddler OR child* OR preschool OR adolescent OR teenager OR "young adult" OR "lowest income group")	Synonyms for population group from scoping search [1,2,3,4]<br><br>Lowest income group and young adult added as terms to reflect key words of two scoping papers [3,4]<br><br>Quotation marks added so that the database recognises it is a phrase and not two separate words, reducing the number of results
Intervention terms	S2	TITLE-ABS-KEY("food insecur*" or "food secur*" or "food poverty" or "food insufficien*" or "food assistance" or "food depriv*" or "poverty" or "foodbanks" or "food banks" or "hunger")	Key terms/synonyms from scoping search [2-6]
Outcome terms	S3	TITLE("nutrition" or "food practices" or "food preferences" or "healthy eating" or "feeding behaviour" or "feeding practices" or "food habits" or "diet" or "diet quality" or "portion size" or "breastfeeding" or "complimentary feeding" or "weaning" or "maternal nutrition" or "child nutrition" or "infant food")	Key terms/synonyms of outcomes of this review [3-7]
	S4	TITLE("weight" or "obesity" or "childhood obesity" or "development" or "growth")	Key terms for nutrition-related health outcomes of this review
	S5	S3 or S4	
Study Design terms	S6	"qualitative research" or "grounded theory" or "ethnograph*" or "phenomenolog*" or "feminis*" or "narrative*" or "interview*" or "focus group*" or "case stud*" or "anthrop*" or "observ*" or "field notes" or "biograph*" or "life history" or "photovoice" or "photo elicitation" or "autoethnograph*" or "creative method" or "thematic analysis"	Refine results by study design as this is a qualitative systematic review
	S7	S1 and S2 and S5 and S6	

		(LIMIT-TO (PUBYEAR,2021) OR LIMIT-TO (PUBYEAR,2020) OR LIMIT-TO (PUBYEAR,2019) OR LIMIT-TO (PUBYEAR,2018) OR LIMIT-TO (PUBYEAR,2017) OR LIMIT-TO (PUBYEAR,2016) OR LIMIT-TO (PUBYEAR,2015) OR LIMIT-TO (PUBYEAR,2014) OR LIMIT-TO (PUBYEAR,2013) OR LIMIT-TO (PUBYEAR,2012) OR LIMIT-TO (PUBYEAR,2011) OR LIMIT-TO (PUBYEAR,2010) OR LIMIT-TO (PUBYEAR,2009) OR LIMIT-TO (PUBYEAR,2008)	Further refined the results on the basis that this protocol is set within the context of the last 12 years

		(LIMIT-TO (AFFILCOUNTRY , "United States") OR LIMIT-TO (AFFILCOUNTRY , "United Kingdom") OR LIMIT-TO (AFFILCOUNTRY , "Canada") OR LIMIT-TO (AFFILCOUNTRY , "Australia") OR LIMIT-TO (AFFILCOUNTRY , "Germany") OR LIMIT-TO (AFFILCOUNTRY , "France") OR LIMIT-TO (AFFILCOUNTRY , "Italy") OR LIMIT-TO (AFFILCOUNTRY , "Netherlands") OR LIMIT-TO (AFFILCOUNTRY , "Switzerland") OR LIMIT-TO (AFFILCOUNTRY , "Spain") OR LIMIT-TO (AFFILCOUNTRY , "Sweden") OR LIMIT-TO (AFFILCOUNTRY , "Norway") OR LIMIT-TO (AFFILCOUNTRY , "New Zealand") OR LIMIT-TO (AFFILCOUNTRY , "Japan") OR LIMIT-TO (AFFILCOUNTRY , "Belgium") OR LIMIT-TO (AFFILCOUNTRY , "Denmark") OR LIMIT-TO (AFFILCOUNTRY , "Finland") OR LIMIT-TO (AFFILCOUNTRY , "Poland") OR LIMIT-TO (AFFILCOUNTRY , "Chile") OR LIMIT-TO (AFFILCOUNTRY , "Austria") OR LIMIT-TO (AFFILCOUNTRY , "Ireland") OR LIMIT-TO (AFFILCOUNTRY , "Israel") OR LIMIT-TO (AFFILCOUNTRY , "Portugal") OR LIMIT-TO (AFFILCOUNTRY , "Czech Republic") OR LIMIT-TO (AFFILCOUNTRY , "Greece") OR LIMIT-TO (AFFILCOUNTRY , "Hungary") OR LIMIT-TO (AFFILCOUNTRY , "Lithuania") OR LIMIT-TO (AFFILCOUNTRY , "Luxembourg") OR LIMIT-TO (AFFILCOUNTRY , "Slovakia") OR LIMIT-TO (AFFILCOUNTRY , "Estonia") OR LIMIT-TO (AFFILCOUNTRY , "Iceland") OR LIMIT-TO (AFFILCOUNTRY , "Slovenia") OR LIMIT-TO (AFFILCOUNTRY , "Latvia") OR LIMIT-TO (AFFILCOUNTRY , "Undefined")	Refined results to high income countries as defined by the OECD members list[8]<br><br>This was not added as a concept into the search strategy because of the large number of alternative words associated with a country that could be in the title or abstract. For instance, England / English (Engl*) or Holland / Dutch.<br><br>Another reason was because in the UK, for instance, the existing qualitative evidence base is limited to cities and regions, so country name might not be in the title [9]<br><br>Scoping showed that limiting the search by affiliate country did not exclude key papers
		(LIMIT-TO (LANGUAGE , "English")	Limited to English language

Limit justification:

This search was limited to the last 12 years to set the review because because post financial crises all high-income countries suffered an economic crash, alongside increasing poverty rates and food insecurity

The search was further refined to English language or existing translation in English due to project time constraints and budget. It was limited to human participants given that this is the population of interest.

1 Attree, P. (2004), Growing up in disadvantage: a systematic review of the qualitative evidence. Child: Care, Health and Development, 30: 679-689.

2 Attree, P. (2005), Low-income mothers, nutrition and health: a systematic review of qualitative evidence. Maternal & Child Nutrition, 1: 227-240.

3 Gross RS, Mendelsohn AL, Arana MM, Messito MJ. (2019) Food Insecurity During Pregnancy and Breastfeeding by Low-Income Hispanic Mothers. Pediatrics, 143(6)

4 Harden, J. and Dickson, A. (2015) 'Low-income mothers' food practices with young children: A qualitative longitudinal study', Health Education Journal, 74(4), pp. 381–391

5 Frank, L. (2015) Exploring Infant Feeding Practices In Food Insecure Households: What Is The Real Issue?, Food and Foodways, 23:3, 186-209

6 Lovelace S, Rabiee-Khan, F. (2015) Food choices made by low-income households when feeding their pre-school children: a qualitative study, Maternal Child Nutrition, 11(4), pp. 870-81

7 Zorbas C, Palermo C, Chung A, Iguacel I, Peeters A, Bennett R, Backholer K. (2018) Factors perceived to influence healthy eating: a systematic review and meta-ethnographic synthesis of the literature, Nutrition Reviews, 76(12) pp. 861–874

8 OECD. List of OECD High-Income Economies. Available at: https://www.worldbank.org/en/news/press-release/2019/10/24/doing-business-2020-oecd-high-income-economies-remain-global-benchmarks-on-most-doing-business-indicators. Accessed November, 2020.

9 Puddephatt J-A, Keenan GS, Fielden A, Reaves DL, Halford JCG, Hardman CA. (2020) 'Eating to survive': A qualitative analysis of factors influencing food choice and eating behaviour in a food-insecure population. Appetite, 147:104547.
