# Supplementary material for: Food insecurity and the nutritional health and well-being of women and children in high-income countries: protocol for a qualitative systematic review
Source: BMJ Open. 2021 Aug 26;11(8):e048180. doi: 10.1136/bmjopen-2020-048180 (PMC8395272; doi:10.1136/bmjopen-2020-048180)
Supplement: Author's manuscript [file bmjopen-2020-048180.draft_revisions.pdf]

BMJ Open

BMJ Open is committed to open peer review. As part of this commitment we make the peer review history of every article we publish publicly available.

When an article is published we post the peer reviewers' comments and the authors' responses online. We also post the versions of the paper that were used during peer review. These are the versions that the peer review comments apply to.

The versions of the paper that follow are the versions that were submitted during the peer review process. They are not the versions of record or the final published versions. They should not be cited or distributed as the published version of this manuscript.

BMJ Open is an open access journal and the full, final, typeset and author-corrected version of record of the manuscript is available on our site with no access controls, subscription charges or pay-per-view fees (http://bmjopen.bmj.com).

If you have any questions on BMJ Open's open peer review process please email info.bmjopen@bmj.com

BMJ Open

Food insecurity and the nutritional health and wellbeing of women and children in high-income countries: protocol for a qualitative systematic review

Journal:	*BMJ Open*
Manuscript ID	bmjopen-2020-048180
Article Type:	Protocol
Date Submitted by the Author:	21-Dec-2020
Complete List of Authors:	Bell, Zoe; Newcastle University Faculty of Medical Sciences, Population Health Sciences Institute<br>Scott, Steph; Newcastle University Faculty of Medical Sciences, Population Health Sciences Institute<br>Visram, Shelina; Newcastle University Faculty of Medical Sciences, Population Health Sciences Institute<br>Rankin, Judith; Newcastle University, Population Health Sciences Institute<br>Bambra, Clare; Newcastle University<br>Heslehurst, Nicola; Newcastle University
Keywords:	QUALITATIVE RESEARCH, NUTRITION & DIETETICS, PUBLIC HEALTH

SCHOLARONE™
Manuscripts

I, the Submitting Author has the right to grant and does grant on behalf of all authors of the Work (as defined in the below author licence), an exclusive licence and/or a non-exclusive licence for contributions from authors who are: i) UK Crown employees; ii) where BMJ has agreed a CC-BY licence shall apply, and/or iii) in accordance with the terms applicable for US Federal Government officers or employees acting as part of their official duties; on a worldwide, perpetual, irrevocable, royalty-free basis to BMJ Publishing Group Ltd ("BMJ") its licensees and where the relevant Journal is co-owned by BMJ to the co-owners of the Journal, to publish the Work in this journal and any other BMJ products and to exploit all rights, as set out in our licence.

The Submitting Author accepts and understands that any supply made under these terms is made by BMJ to the Submitting Author unless you are acting as an employee on behalf of your employer or a postgraduate student of an affiliated institution which is paying any applicable article publishing charge ("APC") for Open Access articles. Where the Submitting Author wishes to make the Work available on an Open Access basis (and intends to pay the relevant APC), the terms of reuse of such Open Access shall be governed by a Creative Commons licence – details of these licences and which Creative Commons licence will apply to this Work are set out in our licence referred to above.

Other than as permitted in any relevant BMJ Author's Self Archiving Policies, I confirm this Work has not been accepted for publication elsewhere, is not being considered for publication elsewhere and does not duplicate material already published. I confirm all authors consent to publication of this Work and authorise the granting of this licence.

For peer review only

Food insecurity and the nutritional health and wellbeing of women and children in high-income countries: protocol for a qualitative systematic review

Zoë Bell*[1], Steph Scott[2], Shelina Visram[3], Judith Rankin[4], Clare Bambra[5], Nicola Heslehurst [1]

*Corresponding author: Zoë Bell: z.bell2@ncl.ac.uk

[1] nicola.heslehurst@newcastle.ac.uk Population Health Sciences Institute, Newcastle University, Newcastle upon Tyne, NE1 4LP

[2] steph.scott@newcastle.ac.uk Population Health Sciences Institute, Newcastle University, Newcastle upon Tyne, NE1 4LP

[3] shelina.visram@newcastle.ac.uk Population Health Sciences Institute, Newcastle University, Newcastle upon Tyne, NE1 4LP

[4] judith.rankin@newcastle.ac.uk Population Health Sciences Institute, Newcastle University, Newcastle upon Tyne, NE1 4LP

[5] clare.bambra@newcastle.ac.uk Population Health Sciences Institute, Newcastle University, Newcastle upon Tyne, NE1 4LP

Keywords: Food insecurity, Nutrition, Qualitative research, Systematic review

Word count: 3,402 excluding references, title page, figures and tables

For peer review only

Abstract

Introduction. Since the global financial crises of 2008, there has been a rise in the number of people experiencing food insecurity. The COVID-19 pandemic has exacerbated this, with many more unable to afford or access food of sufficient quality and quantity to enable good health and wellbeing. Particularly vulnerable are mothers with young children, pregnant women, and lone parents (the majority of whom are women). This review aims to understand experiences of food insecurity and how it affects women and children's nutritional health and wellbeing. Findings will help guide health policy and practice to support food insecure women and children from high-income countries.

Methods and analysis. A systematic review and meta-ethnography exploring i) food insecure women's own accounts of their nutritional health and ii) food insecure household's accounts of their children's nutritional health will be conducted. Six major databases (Medline, Scopus, Web of Science, EMBASE, CINAHL and ASSIA), will be searched from 1st January 2008 to 30th March 2021. Grey literature databases and relevant stakeholder websites will also be searched. Electronic database searches will be supplemented by reference list and citation searches. Outcomes of interest are accounts of nutrition and nutritional health, including diet, food practices, infant feeding practices, physical and mental health. The review will be carried out using the Preferred Reporting Items of Systematic Reviews and Meta-Analyses Protocol (PRISMA) guidelines, but as this is a meta-ethnography we will adhere to eMERGe Reporting Guidance for synthesis and writing findings of the final report. The quality of studies will be assessed using the Critical Appraisal Skills Programme (CASP) qualitative checklist. A meta-ethnographic analysis will be conducted for all included studies.

Ethics and dissemination: As this will be a qualitative systematic review, without primary data collection, ethical approval will not be required. Findings will be submitted for peer-reviewed publication.

In accordance with the guidelines, our systematic review protocol was registered with PROSPERO on 23rd October 2020 (registration number CRD42020214159).

Strengths and Limitations of this study

- Employing a rigorous international gold standard methodology (PRISMA-P) to facilitate the development of this protocol and review conduct (PRISMA)
- Adherence to eMERGe Reporting Guidance for synthesising and writing-up the findings of the review
- Comprehensive broad search strategy, supplemented with reference and citation searches
- A potential limitation of the review is that studies will be from different countries with different welfare states, social security, food aid and healthcare systems, which may impact upon temporal relevancy of findings.

For peer review only

Introduction

The terms food insecurity and food poverty both signify "the inability to consume an adequate quality or sufficient quantity of food in socially acceptable ways, or the uncertainty that one will be able to do so" (1). In high-income countries (HICs), food insecurity is generally not the result of supply failures but retrenchment of welfare states and diminishing rights to access healthy food (2-5). The financial crises of 2008 meant economic downturn for all HICs, with countries responding differently to the crises. However, since 2008, poverty rates have increased and reports have consistently documented increased use of foodbanks, a proxy measure of food insecurity (6, 7). The COVID-19 pandemic has worsened food insecurity which was already an urgent public health issue (8). Governments imposed, to varying degrees, lockdown measures that increased social isolation and confinement within the home to reduce the reproduction rate of the virus (9). The effects are likely to be unequal across society and are expected to lead to higher poverty rates, having profound effects on economically vulnerable families (10). Families who were teetering on the edge of just managing may be pushed into food insecurity, thereby experiencing hunger, reduced food consumption, and creating an inability to secure food of sufficient quality and quantity to enable good health and participation in society. Particularly vulnerable are families with young children, pregnant women and lone parents, the majority of which are women (11).

It is well established that a healthy balanced diet is an important factor for health. Preconception health is strongly linked not only to pregnancy outcomes, but life course research evidences this period as crucial for health across generations (12), widely recognised is the importance of nutrition during the first 1000 days of life (conception, pregnancy to two years of age) (13). As the diet of a young child is largely determined by the mother, understanding how food insecurity effects women's nutritional health and wellbeing is important. To date, most reviews exploring the effects of food insecurity on nutrition have reviewed quantitative studies (14-17). Quantitative analyses reveal that individuals experiencing food insecurity are more likely to have a poorer quality diet than those who are food secure (18). Food insecurity is adversely associated with dietary quality for adults; consuming fewer vegetables, fruit, and dairy products than food secure adults and having lower intake of vitamins A, B-6, calcium, magnesium, iron and zinc (15). For children, food insecurity is less consistently associated with lower dietary quality although there is substantial evidence of lower intake of fruit and vegetables (15). Factors influencing the relationship between food insecurity and diet quality remains unclear.

Quantitative studies also report on nutrition-related health outcomes, food insecurity being associated with many non-communicable diseases (14, 19). A major public health concern is the robust association of food insecurity with obesity amongst children and adults in HICs (14, 19-22). Substantial risks for maternal and child health are associated with mothers who are overweight (12). Explanations for the relationship between food insecurity and nutrition-related health remain unclear. Proposed theoretical explanations include sacrifice theory where mothers forego food for their child (supported by the strong relationship between food insecurity and obesity for mothers), substitution hypothesis whereby nutrient-dense, low-energy foods are substituted for cheaper, energy-dense often higher fat and sugar-containing foods (23) and the insurance hypothesis associated with the cyclical nature of food insecurity and its disruption on metabolism (24). Emotional distress has also been theorised as important in the relationship between food insecurity and nutrition-related health (25, 26).

Qualitative research can provide deeper insight into this area. A body of qualitative evidence is accumulating documenting women and children's experiences and perceptions of food insecurity in relation to their nutritional health and wellbeing. Food insecure participants report altered diets via restrictive eating patterns, for example, reducing portion size, skipping meals or forgoing an entire day of food (27-29). Food insecure participants also report worsened health issues, such as stress, depression and weight gain, which has a knock-on negative effect for the health and wellbeing of dependent children (27, 30) (31). Breastfeeding is the healthiest method for infant feeding, yet a paradox exists where infants from lower-income households are least likely to be breastfed, or for as long, despite arguably gaining most from the health and cost benefits it

For peer review only

provides (32, 33). Reviewing qualitative research will help to develop an understanding of the complexities around food insecurity and nutritional health and wellbeing.

Furthermore, despite most health research for children and young people being based on parent's, caregivers or stakeholders' views, there is evidence that parents are not always aware of their children's experiences of food insecurity (34). Studies have reported inconsistencies between parent's versus children's perspectives of what the child is experiencing in the household (34). Indeed, evidence shows that children experience food insecurity in different ways to adults (35-37). Systematically reviewing the literature for family unit's perspectives of the effects on food insecurity on children's nutritional health and wellbeing is important to explore the differences and gain a holistic view of it effect.

The aim of this qualitative systematic review is to explore food insecure women's and children's experiences of nutritional health and wellbeing in HICs, within the context of the last 12 years. This review will further our understanding, inform health policy and practice, and result in recommendations for researchers and areas for further research.

Methods

Preliminary searches were carried out in September 2020 and the study registered with PROSPERO (38) on 23rd October 2020 (registration number CRD42020214159). The review started November 2020 and is anticipated to take eleven months to complete.

The review will use meta-ethnography, one of the most developed and structured methods to synthesise qualitative findings, developed by Noblit and Hare (1988) (39). Meta-ethnography involves a seven-phase approach: 1. Getting started 2. Deciding what is relevant to the initial interest 3. Reading the studies 4. Determining how the studies are related 5. Translating the studies into one another 6. Synthesising translations 7. Expressing the synthesis.

Review questions

1. How do women perceive food insecurity to affect their nutrition and nutritional health?
2. How do food insecure household's perceive food insecurity to affect their child's nutrition and nutritional health?

Objectives

The study objectives are:

1. To explore food insecure women's experiences and accounts of their own nutritional health
2. To explore food insecure household's accounts of their children's nutritional health experiences

Eligibility criteria

Studies will be selected according to the inclusion and exclusion criteria outlined in table 1. Eligibility criteria are outlined in accordance with the modified PICO tool, PICOS, which is deemed appropriate for use in qualitative evidence reviews (40, 41).

Table 1 Eligibility Criteria for screening of studies

	**Inclusion**	**Exclusion**
**Participant (P)**	Food insecure women of childbearing age of all ethnicities (objective one)	Studies restricted to a specific type of population not directly related to women and children/ wider population with clinical needs, that necessitates a specific diet (e.g.

For peer review only

	Food insecure households (parents, primary caregivers, children) of all ages and ethnicities (objective two)	studies in the context of people living with HIV, type 1 diabetes etc.)<br><br>Studies based on university campus with college students (unless in the context of also being a parent)<br><br>Perspectives of those outside the household family unit i.e. grandparents, healthcare professionals, teachers or food programme coordinators (Objective Two)
**Intervention (I) or Exposure**	Food insecurity.<br><br>Other terms used to describe food insecurity and included are for example food poverty, food deprivation, food insufficiency, hunger.	Food secure population groups<br><br>Studies that were qualitative process evaluations of food insecurity-related interventions/services and focused on women and/or children
**Comparison (C)**	Not applicable – systematic review of qualitative studies	
**Outcomes (O)**	Experiences and accounts of the effect of food insecurity on nutrition and nutritional health and wellbeing	Experiences and accounts not explicitly related to food.
**Study type**	Qualitative studies of any design including but not limited to:<br><br>Ethnography, interviews, focus groups, photo elicitation, visual techniques, phenomenology, grounded theory, case study, feminist research, action research.<br><br>Mixed method studies	Quantitative studies<br><br>Reviews<br><br>Expert opinion articles<br><br>Editorials<br><br>Policy documents

For peer review only

	Primary data sources from grey literature and relevant stakeholder websites	Conference abstracts<br><br>Qualitative research that reports no lay perspectives but has analysed text i.e. discourse analysis<br><br>Grey literature that does not include primary qualitative data
**Study period**	Published in the last 12 years (1 Jan 2008- 2021)<br><br>Studies with data collected from 2008 onwards	Literature published before 1 Jan 2008<br><br>Studies reporting data only collected before 2008
**Setting**	High-income countries (as per OECD definition, see supplementary file A)	Non-high-income countries
**Study reporting language**	English	

For peer review only

Exposure

This review will explore the experiences and accounts of those who are food insecure. Food insecurity is defined as 'the inability to consume an adequate quality or sufficient quantity of food in socially acceptable ways, or the uncertainty that one will be able to do' (1). Other terms used to describe food insecurity are included, for example food poverty, food deprivation, food insufficiency and hunger. In this review, we will include those who are experiencing life on low-income, in receipt of income benefit, accessing food aid and those accessing food through public health programmes for example WIC, SNAP and Healthy Start Vouchers.

Outcomes

Primary

Studies will be included if they report experiences and accounts relating to the effect of food insecurity on nutrition and nutritional health. In the context of this review, nutrition outcomes can be reported as accounts of diet (quality and quantity of food, eating behaviour, eating pattern), food practices (i.e. food acquisition, food preparation, organisation and storage of food in the house) and infant feeding practices (breastfeeding, infant formula and complimentary feeding behaviour). Nutritional health and wellbeing outcomes for women and children include physical (e.g. perspectives on their weight or growth and development of a child) and mental (e.g. anxiety about household food running out).

Studies exploring food insecure women and children's health more broadly will not be included unless the health effects are explicitly linked to nutrition. Examples of non-nutrition related health effects of food insecurity include stress, anxiety or depression experienced in the context of being unable to pay the household bills or generally living in poverty where there is no mention of nutrition.

Study exclusion

Studies will be excluded if they were published prior to 2008. The year 2008 was selected as a start-date because this marks the beginning of the global financial crises, since which there has been a rise in the number of people experiencing food insecurity. Studies will be excluded if data collection took place prior to 2008. This is to capture the experiences of food insecurity post-global financial crises. Only HICs will be included. See Supplementary File A for a list of HICs as per OECD high-income economies definition (42).

Study design

Qualitative studies of any design will be included if they report experiences and accounts of food insecure women (objective 1) and / or experiences and accounts of household (parents, primary caregivers, children, objective 2) nutrition and nutritional health. Mixed method studies will be included if qualitative data can be extracted independently from quantitative data. Primary data sources from grey literature relevant stakeholder websites will be included if they report primary qualitative data relating to the review.

Search Strategy

We will search six bibliographic databases: MEDLINE, EMBASE, CINAHL, Scopus, Applied Social Science Index and Abstracts (ASSIA) and Web of Science from 1st January 2008 until 30th March 2021. Theses and dissertations will be searched using Open Access Theses and Dissertations (OATD). Grey literature will be searched using OpenGrey Europe (for information on Grey Literature in Europe) and Trove (links to Australian grey literature). Relevant stakeholder websites will be searched in March 2021 (see Supplementary File B). Only publications in English language will be included.

Search terms were identified from literature within the field, and the search strategy has been designed and piloted with an information scientist at Newcastle University. The strategy consists of four main concepts in accordance with the PICOS tool (41) (table 1). An example search strategy for Scopus can be found in Supplementary File C.

The descriptive titles of qualitative studies often lead to inappropriate indexing, posing challenges in finding relevant studies when searching bibliographic databases alone (43). For this reason, we will screen included studies reference lists for other studies published prior to the included study and use citation searching for studies citing the included study, using Google Scholar.

Study Selection

Studies will be imported into EndNote version X9.3.3 (44)for de-duplication, then imported into Rayyan (45) an online program for systematic reviews. All titles and abstracts will be screened by ZB and a second reviewer (split between SS, SV and NH). Full texts will also be double screened. A pilot exercise screening 30 titles and abstracts will be carried out across the screening team (ZB, SS, SV) to calibrate and test the full-text review form. A third reviewer will assist to resolve any disagreements. Reasons for exclusion at the full-text stage will be recorded and a PRISMA flowchart will be used to report each stage of screening.

A standerdised data extraction table will be created as a data extraction form and piloted using a sub-set of included studies. The following key information will be extracted from included studies: aim(s), country, time period, population characteristics (e.g. who the accounts and experiences are from i.e. women, pregnant women or breastfeeding, parents, caregivers or children, age ranges, income/employment/SES), sample size, study methods, summary of the main themes with some exemplary direct quotes and context of the research. This information will form a table of included studies. Data will be extracted by one reviewer (ZB).

Risk of bias assessment

Quality of included studies will be assessed using the Critical Appraisal Skills Programme (CASP) qualitative checklist (46). The checklist covers the primary focus of the paper, appropriateness of the study design, sample recruitment, methodology, analysis and generalisability. Quality appraisal will be undertaken by one

For peer review only

reviewer (ZB), a sample of included studies will be double reviewed for quality appraisal to check agreement. CASP appraisals will be used to inform the data synthesis stage and provide an overview of the quality of included studies for context and inform discussion of the strengths and limitations of existing evidence.

Data synthesis

Methods for review synthesis are to be determined through informed conversations of the nature of the evidence available, the review questions and purpose (47). Meta-ethnography places studies side by side to see how key themes can be translated between studies whilst considering similarities and differences across varied contexts. This interpretive approach moves beyond describing or aggregating findings, instead aiming to 'synthesise understanding' (39). If required, to 'synthesise understanding' we will contact authors of studies to check interpretations of important points (48). The integration of findings of multiple studies will enable the development of deeper insights into the understanding of food insecurity in the context of nutrition, which individual studies alone cannot provide.

Meta-ethnography synthesis will be conducted in seven steps using NVivo 10 software (49). Step one, in-depth reading of included studies by three reviewers (ZB, SS and SV). Step two, creation of study sub-sets and line-by-line coding and extracting of first and second order themes. The third step is to determine how studies are related. We will tabulate first order themes (interpretations) and second order themes (interpretation of interpretations) with grouped studies to create 'meta-themes'. The fourth and fifth steps will involve translating studies by checking first and second order concepts and themes against each other. They may be similar (reciprocal) or refutational. Step six is synthesising the translations to create a third order construct. Step seven is expressing the synthesis through dissemination.

A sample of papers will be duplicate-coded and discussed with the review team. This is to view the data through different perspectives (i.e. a form of investigator triangulation) rather than to check for consistency in coding between reviewers, which is a more positivist approach. ZB will analyse and synthesise the entire dataset. When synthesising and writing-up the findings of the review, we will adhere to eMERGe Reporting Guidance, recommended to ensure complete and transparent reporting of meta-ethnographies (50).

Patient and Public Involvement

Patients and members of the public were not involved in the design of this protocol. We will consult with local community members for dissemination of the review findings.

Discussion

To the best of our knowledge, this is the first systematic review which aims to synthesize and interpret the findings of qualitative studies of food insecurity in relation to nutritional health, focusing on women and children from HICs. We believe it is important to set the study within the context of the last 12 years as, post-financial crisis, all HICs suffered an economic crash alongside increasing poverty rates. Excluding studies with data collected prior to 2008 ensures that the synthesis is contextualized by the effects of the last financial crises and events since, such as the COVID-19 pandemic.

We recognize that for HICs outside of the USA and Canada qualitative literature in this area is limited. In response to this we have taken a comprehensive approach to our search strategy in terms of searching stakeholder websites to locate relevant grey literature that might contain primary qualitative data. This is supplemented by screening the reference lists and citations of included studies. To maximize rigor and transparency, this qualitative systematic review was designed following established protocols, and will adhere to recommended and validated methods and reporting guidelines.

A potential limitation of the review is that it will include studies from multiple countries. It may be challenging to draw meaningful conclusions from studies involving different welfare states, social security, food aid and healthcare systems (51, 52). However, the study is set within the context of the global financial

For peer review only

crises and not the response to this. Deciding to limit the studies to English language only might exclude some relevant studies, particularly from European countries. However, due to funding and time limitations, translation is beyond the scope of this review.

This synthesis is relevant because it is set within the context of the last financial crises and more recently, a global pandemic that is beginning to result in further economic downturn. These world events have the potential to exacerbate health inequalities. It is important that research focusing on health inequalities is shaped by understandings from people who are experiencing them (53, 54). This review does that by aiming to further our understanding of the experiences of food insecure women and children in the context of nutrition. This review will therefore provide valuable insights for future research and help guide health policy and practice to support food insecure women and children from HICs.

Ethics and Dissemination

The review is using secondary data, so ethical approval is not required. Review findings will be disseminated by publication in theses, peer-reviewed academic journal articles, conferences, policy and practice workshops such as those organized by Fuse: the UK Centre for Translational Research in Public Health (www.fuse.ac.uk) and circulated to the general public and stakeholder groups using social media.

Author Contributions

ZB drafted the manuscript and is the guarantor for the review. All authors contributed to the development of the selection criteria. ZB, NH, SS, SV contributed to the quality appraisal assessment strategy and data extraction criteria. ZB developed the search strategy. All authors provided comments and amendments. All authors read, provided feedback and approved the final manuscript.

Funding Statement

This systematic review was supported by the Economic and Social Research Council (ESRC), as part of a doctoral research project. No award or grant number received. The funder had no role in developing the systematic review protocol and will not be involved in conducting the review.

Conflicts of interest

None declared.

Abbreviations

HIC: High-income countries

OECD: Organisation for Economic Co-Operation and Development

PRISMA: Preferred Reporting Items for Systematic Reviews and Meta-Analyses

PRISMA-P: Preferred Reporting Items for Systematic Reviews and Meta-Analyses (for Protocols)

PROSPERO: International prospective register of systematic reviews

SNAP: Supplemental Nutrition Assistance Program

WIC: Supplemental Nutrition Assistance Program for Women, Infants and Children

For peer review only

Bibliography

1. Dowler E. Food and Poverty in Britain: Rights and Responsibilities. Social Policy & Administration. 2002;36(6):698-717.
2. Dowler EA, Kneafsey M, Lambie H, Inman A, Collier R. Thinking about 'food security': engaging with UK consumers. Critical Public Health. 2011;21(4):403-16.
3. Dowler EA, O'Connor D. Rights-based approaches to addressing food poverty and food insecurity in Ireland and UK. Social Science & Medicine. 2012;74(1):44-51.
4. Riches G, Silvasti T. Hunger in the Rich World: Food Aid and Right to Food Perspectives. 2014.
5. Lambie-Mumford H. The growth of food banks in Britain and what they mean for social policy. Critical Social Policy. 2018;39(1):3-22.
6. Davis O, Geiger BB. Did Food Insecurity rise across Europe after the 2008 Crisis? An analysis across welfare regimes. Social Policy and Society. 2017;16(3):343-60.
7. Lambie-Mumford H, Green MA. Austerity, welfare reform and the rising use of food banks by children in England and Wales. Area. 2017;49(3):273-9.
8. Loopstra R. Vulnerability to food insecurity since the COVID-19 lockdown The Food Foundation Kings College London; 2020.
9. Bambra C, Riordan R, Ford J, Matthews F. The COVID-19 pandemic and health inequalities. Journal of Epidemiology and Community Health. 2020;74(11):964.
10. International Labour Organisation. The impact of the COVID-19 pandemic on jobs and incomes in G20 economies. 2020.
11. Annesley C, Himmelweit S. The Impact on Women of the Coalition Spending Review 2010. 2010.
12. Stephenson J, Heslehurst N, Hall J, Schoenaker DAJM, Hutchinson J, Cade JE, et al. Before the beginning: nutrition and lifestyle in the preconception period and its importance for future health. The Lancet. 2018;391(10132):1830-41.
13. Barker DJP. The origins of the developmental origins theory. Journal of Internal Medicine. 2007;261(5):412-7.
14. Gundersen C, Ziliak JP. Food Insecurity And Health Outcomes. Health Affairs. 2015;34(11):1830-9.
15. Hanson KL, Connor LM. Food insecurity and dietary quality in US adults and children: a systematic review. Am J Clin Nutr. 2014;100(2):684-92.
16. Myers CA, Mire EF, Katzmarzyk PT. Trends in Adiposity and Food Insecurity Among US Adults. JAMA Network Open. 2020;3(8):e2012767-e.
17. de Oliveira KHD, de Almeida GM, Gubert MB, Moura AS, Spaniol AM, Hernandez DC, et al. Household food insecurity and early childhood development: Systematic review and meta-analysis. Matern Child Nutr. 2020;16(3):e12967-e.
18. Yang TC, Sahota P, Pickett KE, Bryant M. Association of food security status with overweight and dietary intake: exploration of White British and Pakistani-origin families in the Born in Bradford cohort. Nutrition Journal. 2018;17(1):48.
19. Larson NI, Story MT. Food insecurity and weight status among U.S. children and families: a review of the literature. Am J Prev Med. 2011;40(2):166-73.
20. Townsend MS, Peerson J, Love B, Achterberg C, Murphy SP. Food Insecurity Is Positively Related to Overweight in Women. The Journal of Nutrition. 2001;131(6):1738-45.
21. Dinour LM, Bergen D, Yeh MC. The food insecurity-obesity paradox: a review of the literature and the role food stamps may play. J Am Diet Assoc. 2007;107(11):1952-61.
22. Franklin B, Jones A, Love D, Puckett S, Macklin J, White-Means S. Exploring mediators of food insecurity and obesity: a review of recent literature. J Community Health. 2012;37(1):253-64.
23. Drewnowski A. Obesity and the food environment: dietary energy density and diet costs. Am J Prev Med. 2004;27(3 Suppl):154-62.
24. Nettle D, Andrews C, Bateson M. Food insecurity as a driver of obesity in humans: The insurance hypothesis. Behav Brain Sci. 2017;40:e105.
25. Hemmingsson E. A new model of the role of psychological and emotional distress in promoting obesity: conceptual review with implications for treatment and prevention. Obesity Reviews. 2014;15(9):769-79.
26. Hemmingsson E. Early Childhood Obesity Risk Factors: Socioeconomic Adversity, Family Dysfunction, Offspring Distress, and Junk Food Self-Medication. Curr Obes Rep. 2018;7(2):204-9.

For peer review only

27. Harvey K. "When I go to bed hungry and sleep, I'm not hungry": Children and parents' experiences of food insecurity. Appetite. 2016;99:235-44.
28. Puddephatt J-A, Keenan GS, Fielden A, Reaves DL, Halford JCG, Hardman CA. 'Eating to survive': A qualitative analysis of factors influencing food choice and eating behaviour in a food-insecure population. Appetite. 2020;147:104547.
29. Purdam K, Garratt EA, Esmail A. Hungry? Food Insecurity, Social Stigma and Embarrassment in the UK. Sociology. 2015;50(6):1072-88.
30. Garthwaite K, Collins PJ, Bambra C. Food for Thought: An Ethnographic Study of Negotiating Ill Health and Food Insecurity in a UK Foodbank. Social science & medicine (1982). 2015;132:38-44.
31. Thompson C, Smith D, Cummins S. Understanding the health and wellbeing challenges of the food banking system: A qualitative study of food bank users, providers and referrers in London. Social Science & Medicine. 2018;211:95-101.
32. Venu I, van den Heuvel M, Wong JP, Borkhoff CM, Moodie RG, Ford-Jones EL, et al. The breastfeeding paradox: Relevance for household food insecurity. Paediatr Child Health. 2017;22(4):180-3.
33. van den Heuvel M, Birken C. Food insecurity and breastfeeding. Cmaj. 2018;190(11):E310-e1.
34. Fram MS, Frongillo EA, Jones SJ, Williams RC, Burke MP, DeLoach KP, et al. Children are aware of food insecurity and take responsibility for managing food resources. J Nutr. 2011;141(6):1114-9.
35. Attree P. The Social Costs of Child Poverty: A Systematic Review of the Qualitative Evidence. Children & Society. 2006;20(1):54-66.
36. Ridge T. The Everyday Costs of Poverty in Childhood: A Review of Qualitative Research Exploring the Lives and Experiences of Low-Income Children in the UK. Children & Society. 2011;25(1):73-84.
37. Connell CL, Lofton KL, Yadrick K, Rehner TA. Children's experiences of food insecurity can assist in understanding its effect on their well-being. J Nutr. 2005;135(7):1683-90.
38. National Institute for Health Research. International prospective register of systematic reviews 2020 [Available from: https://www.crd.york.ac.uk/prospero/.
39. Noblit G, Hare D. Meta-Ethnography. Thousand Oaks, California1988. Available from: https://methods.sagepub.com/book/meta-ethnography.
40. Methley AM, Campbell S, Chew-Graham C, McNally R, Cheraghi-Sohi S. PICO, PICOS and SPIDER: a comparison study of specificity and sensitivity in three search tools for qualitative systematic reviews. BMC Health Services Research. 2014;14(1):579.
41. Tacconelli E. Systematic reviews: CRD's guidance for undertaking reviews in health care. The Lancet Infectious Diseases. 2010;10(4):226.
42. The World Bank. Doing Business 2020: OECD High-Income Economies Remain Global Benchmarks on Most Doing Business Indicators The World Bank, 2019 [Available from: https://www.worldbank.org/en/news/press-release/2019/10/24/doing-business-2020-oecd-high-income-economies-remain-global-benchmarks-on-most-doing-business-indicators.
43. Atkins S, Lewin S, Smith H, Engel M, Fretheim A, Volmink J. Conducting a meta-ethnography of qualitative literature: Lessons learnt. BMC Medical Research Methodology. 2008;8(1):21.
44. Clarivate Analytics. EndNote X9.3.3 ed.
45. Institutue QCR. Rayyan QCRI. 2020.
46. Singh J. Critical Appraisal Skills Programme. CASP Appraisal Tools. Journal of Pharmacology and Pharmacotherapeutics. 2013;4:76.
47. Booth A. Systematic approaches to a successful literature review. London ;

Thousand Oaks, Calif.:: Sage; 2012.
48. Green J, Thorogood N. Qualitative Methods for Health Research. SAGE. 2004;9:177-80.
49. International Q. NVivo. 11 ed2020.
50. France EF, Uny I, Ring N, Turley RL, Maxwell M, Duncan EAS, et al. A methodological systematic review of meta-ethnography conduct to articulate the complex analytical phases. BMC Medical Research Methodology. 2019;19(1):35.
51. Hillier-Brown F, Thomson K, McGowan V, Cairns J, Eikemo TA, Gil-Gonzále D, et al. The effects of social protection policies on health inequalities: Evidence from systematic reviews. Scandinavian Journal of Public Health. 2019;47(6):655-65.
52. Hamnett C. Shrinking the welfare state: the structure, geography and impact of British government benefit cuts. Transactions of the Institute of British Geographers. 2014;39(4):490-503.

For peer review only

53. Popay J. What will it take to get the evidential value of lay knowledge recognised? International Journal of Public Health. 2018;63(9):1013-4.
54. Popay J, Williams G, Thomas C, Gatrell T. Theorising Inequalities in Health: The Place of Lay Knowledge. Sociology of Health & Illness. 1998;20(5):619-44.

For peer review only

Supplementary File A: List of high-income countries that meet the inclusion criteria (defined by list of OECD High-Income Economies). Available at: https://www.worldbank.org/en/news/press-release/2019/10/24/doing-business-2020-oecd-high-income-economies-remain-global-benchmarks-on-most-doing-business-indicators. Accessed November, 2020)

Australia
Austria
Belgium
Canada
Chile
Czech Republic
Denmark
Estonia
Finland
France
Germany
Greece
Hungary
Iceland
Ireland
Israel
Italy
Japan
Korea
Latvia
Lithuania
Luxembourg
Netherlands
New Zealand
Norway
Poland
Portugal
Slovakia
Slovenia
Spain
Sweden
Switzerland
United Kingdom
United States

For peer review only

Supplementary File B - List of relevant stakeholder websites to be searched

- European Food banks Federation - https://www.eurofoodbank.org/
- Trussell Trust - https://www.trusselltrust.org/
- Independent Food Aid Network - https://www.foodaidnetwork.org.uk/
- The Food Foundation - https://foodfoundation.org.uk/
- Feeding Britain - https://feedingbritain.org/
- Feeding America - https://www.feedingamerica.org/
- Foodbanks Canada - https://www.foodbankscanada.ca/
- Foodbank Australia - https://www.foodbank.org.au/?state=au
- The Foodbank Project - https://www.foodbank.org.nz/
- Royal College of Gynaecology and Obstetrics - https://www.rcog.org.uk/
- Royal College of Nursing - https://www.rcn.org.uk/
- World Health Organiaation and affiliate websites (e.g., UNICEF, International Confederation of Midwives) - https://www.who.int/ https://www.unicef.org.uk/ https://www.internationalmidwives.org/
- Paediatrics; Nursing sub-specialties – Perinatal, Neonatal, Community, Family, Public Health; Midwifery, Dietitian and Doula coalitions and associations – websites from high resourced, Western countries (e.g., International Coalition of Nurses https://www.icn.ch/)
- Baby Friendly Initiative (formerly Baby Friendly Hospital Initiative) websites associated with high resourced, Western countries e.g. https://www.unicef.org.uk/babyfriendly/
- Breastfeeding and Lactation coalitions and associations – websites from high resourced, Western countries (e.g., La Leche League International https://www.llli.org/)

For peer review only

**Search Concept – Scopus**			**Rationale**
Population terms	S1	TITLE-ABS-KEY(wom?n OR female OR mother* OR matern* OR mum OR father OR dad* OR caregiver OR guardian OR parent* OR preg* OR postnatal OR newborn OR baby OR babies OR infan* OR toddler OR child* OR preschool OR adolescent OR teenager OR "young adult" OR "lowest income group")	Synonyms for population group from scoping search [1,2,3,4]<br><br>Lowest income group and young adult added as terms to reflect key words of two scoping papers [3,4]<br><br>Quotation marks added so that the database recognises it is a phrase and not two separate words, reducing the number of results
Intervention terms	S2	TITLE-ABS-KEY("food insecur*" or "food secur*" or "food poverty" or "food insufficien*" or "food assistance" or "food depriv*" or "poverty" or "foodbanks" or "food banks" or "hunger")	Key terms/synonyms from scoping search [2-6]
Outcome terms	S3	TITLE("nutrition" or "food practices" or "food preferences" or "healthy eating" or "feeding behaviour" or "feeding practices" or "food habits" or "diet" or "diet quality" or "portion size" or "breastfeeding" or "complimentary feeding" or "weaning" or "maternal nutrition" or "child nutrition" or "infant food")	Key terms/synonyms of outcomes of this review [3-7]
	S4	TITLE("weight" or "obesity" or "childhood obesity" or "development" or "growth")	Key terms for nutrition-related health outcomes of this review
	S5	S3 or S4	
Study Design terms	S6	"qualitative research" or "grounded theory" or "ethnograph*" or "phenomenolog*" or "feminis*" or "narrative*" or "interview*" or "focus group*" or "case stud*" or "anthrop*" or "observ*" or "field notes" or "biograph*" or "life history" or "photovoice" or "photo elicitation" or "autoethnograph*" or "creative method" or "thematic analysis"	Refine results by study design as this is a qualitative systematic review
	S7	S1 and S2 and S5 and S6	
		(LIMIT-TO (PUBYEAR,2021) OR LIMIT-TO (PUBYEAR,2020) OR LIMIT-TO (PUBYEAR,2019) OR LIMIT-TO (PUBYEAR,2018) OR LIMIT-TO (PUBYEAR,2017) OR LIMIT-TO (PUBYEAR,2016) OR LIMIT-TO (PUBYEAR,2015)	Further refined the results on the basis that this protocol is set within the context of the last 12 years

For peer review only

		OR LIMIT-TO (PUBYEAR,2014) OR LIMIT-TO (PUBYEAR,2013) OR LIMIT-TO (PUBYEAR,2012) OR LIMIT-TO (PUBYEAR,2011) OR LIMIT-TO (PUBYEAR,2010) OR LIMIT-TO (PUBYEAR,2009) OR LIMIT-TO (PUBYEAR,2008)	

For peer review only

		(LIMIT-TO (AFFILCOUNTRY , "United States") OR LIMIT-TO (AFFILCOUNTRY , "United Kingdom") OR LIMIT-TO (AFFILCOUNTRY , "Canada") OR LIMIT-TO (AFFILCOUNTRY , "Australia") OR LIMIT-TO (AFFILCOUNTRY , "Germany") OR LIMIT-TO (AFFILCOUNTRY , "France") OR LIMIT-TO (AFFILCOUNTRY , "Italy") OR LIMIT-TO (AFFILCOUNTRY , "Netherlands") OR LIMIT-TO (AFFILCOUNTRY , "Switzerland") OR LIMIT-TO (AFFILCOUNTRY , "Spain") OR LIMIT-TO (AFFILCOUNTRY , "Sweden") OR LIMIT-TO (AFFILCOUNTRY , "Norway") OR LIMIT-TO (AFFILCOUNTRY , "New Zealand") OR LIMIT-TO (AFFILCOUNTRY , "Japan") OR LIMIT-TO (AFFILCOUNTRY , "Belgium") OR LIMIT-TO (AFFILCOUNTRY , "Denmark") OR LIMIT-TO (AFFILCOUNTRY , "Finland") OR LIMIT-TO (AFFILCOUNTRY , "Poland") OR LIMIT-TO (AFFILCOUNTRY , "Chile") OR LIMIT-TO (AFFILCOUNTRY , "Austria") OR LIMIT-TO (AFFILCOUNTRY , "Ireland") OR LIMIT-TO (AFFILCOUNTRY , "Israel") OR LIMIT-TO (AFFILCOUNTRY , "Portugal") OR LIMIT-TO (AFFILCOUNTRY , "Czech Republic") OR LIMIT-TO (AFFILCOUNTRY , "Greece") OR LIMIT-TO (AFFILCOUNTRY , "Hungary") OR LIMIT-TO (AFFILCOUNTRY , "Lithuania") OR LIMIT-TO (AFFILCOUNTRY , "Luxembourg") OR LIMIT-TO (AFFILCOUNTRY , "Slovakia") OR LIMIT-TO (AFFILCOUNTRY , "Estonia") OR LIMIT-TO (AFFILCOUNTRY , "Iceland") OR LIMIT-TO (AFFILCOUNTRY , "Slovenia") OR LIMIT-TO (AFFILCOUNTRY , "Latvia") OR LIMIT-TO (AFFILCOUNTRY , "Undefined")	Refined results to high income countries as defined by the OECD members list[8]<br><br>This was not added as a concept into the search strategy because of the large number of alternative words associated with a country that could be in the title or abstract. For instance, England / English (Engl*) or Holland / Dutch.<br><br>Another reason was because in the UK, for instance, the existing qualitative evidence base is limited to cities and regions, so country name might not be in the title [9]<br><br>Scoping showed that limiting the search by affiliate country did not exclude key papers
		(LIMIT-TO (LANGUAGE , "English")	Limited to English language

For peer review only

Limit justification:

This search was limited to the last 12 years to set the review because because post financial crises all high-income countries suffered an economic crash, alongside increasing poverty rates and food insecurity

The search was further refined to English language or existing translation in English due to project time constraints and budget. It was limited to human participants given that this is the population of interest.

1 Attree, P. (2004), Growing up in disadvantage: a systematic review of the qualitative evidence. Child: Care, Health and Development, 30: 679-689.

2 Attree, P. (2005), Low-income mothers, nutrition and health: a systematic review of qualitative evidence. Maternal & Child Nutrition, 1: 227-240.

3 Gross RS, Mendelsohn AL, Arana MM, Messito MJ. (2019) Food Insecurity During Pregnancy and Breastfeeding by Low-Income Hispanic Mothers. Pediatrics, 143(6)

4 Harden, J. and Dickson, A. (2015) 'Low-income mothers' food practices with young children: A qualitative longitudinal study', Health Education Journal, 74(4), pp. 381–391

5 Frank, L. (2015) Exploring Infant Feeding Practices In Food Insecure Households: What Is The Real Issue?, Food and Foodways, 23:3, 186-209

6 Lovelace S, Rabiee-Khan, F. (2015) Food choices made by low-income households when feeding their pre-school children: a qualitative study, Maternal Child Nutrition, 11(4), pp. 870-81

7 Zorbas C, Palermo C, Chung A, Iguacel I, Peeters A, Bennett R, Backholer K. (2018) Factors perceived to influence healthy eating: a systematic review and meta-ethnographic synthesis of the literature, Nutrition Reviews, 76(12) pp. 861–874

8 OECD. List of OECD High-Income Economies. Available at: https://www.worldbank.org/en/news/press-release/2019/10/24/doing-business-2020-oecd-high-income-economies-remain-global-benchmarks-on-most-doing-business-indicators. Accessed November, 2020.

9 Puddephatt J-A, Keenan GS, Fielden A, Reaves DL, Halford JCG, Hardman CA. (2020) 'Eating to survive': A qualitative analysis of factors influencing food choice and eating behaviour in a food-insecure population. Appetite, 147:104547.

For peer review only

Supplementary File D: PRIMSA-P 2015 checklist: recommended items to address in a systematic review protocol.

Pe	Item No	Checklist item	Page
**ADMINISTRATIVE INFORMATION**			
Title:			
Identification	1a	Identify the report as a protocol of a systematic review	1
Update	1b	If the protocol is for an update of a previous systematic review, identify as such	n/a
Registration	2	If registered, provide the name of the registry (such as PROSPERO) and registration number	4
Authors:			
Contact	3a	Provide name, institutional affiliation, e-mail address of all protocol authors; provide physical mailing address of corresponding author	1
Contributions	3b	Describe contributions of protocol authors and identify the guarantor of the review	9
Amendments	4	If the protocol represents an amendment of a previously completed or published protocol, identify as such and list changes; otherwise, state plan for documenting important protocol amendments	n/a
Support:			
Sources	5a	Indicate sources of financial or other support for the review	9
Sponsor	5b	Provide name for the review funder and/or sponsor	9
Role of sponsor or funder	5c	Describe roles of funder(s), sponsor(s), and/or institution(s), if any, in developing the protocol	9
**INTRODUCTION**			

For peer review only

Rationale	6	Describe the rationale for the review in the context of what is already known	3-4
Objectives	7	Provide an explicit statement of the question(s) the review will address with reference to participants, interventions, comparators, and outcomes (PICO)	4-5
**METHODS**			
Eligibility criteria	8	Specify the study characteristics (such as PICO, study design, setting, time frame) and report characteristics (such as years considered, language, publication status) to be used as criteria for eligibility for the review	5
Information sources	9	Describe all intended information sources (such as electronic databases, contact with study authors, trial registers or other grey literature sources) with planned dates of coverage	7
Search strategy	10	Present draft of search strategy to be used for at least one electronic database, including planned limits, such that it could be repeated	Supplementary File C
Study records:			
Data management	11a	Describe the mechanism(s) that will be used to manage records and data throughout the review	7-8
Selection process	11b	State the process that will be used for selecting studies (such as two independent reviewers) through each phase of the review (that is, screening, eligibility and inclusion in meta-analysis)	7
Data collection process	11c	Describe planned method of extracting data from reports (such as piloting forms, done independently, in duplicate), any processes for	7

For peer review only

		obtaining and confirming data from investigators	
Data items	12	List and define all variables for which data will be sought (such as PICO items, funding sources), any pre-planned data assumptions and simplifications	7
Outcomes and prioritization	13	List and define all outcomes for which data will be sought, including prioritization of main and additional outcomes, with rationale	6
Risk of bias in individual studies	14	Describe anticipated methods for assessing risk of bias of individual studies, including whether this will be done at the outcome or study level, or both; state how this information will be used in data synthesis	7
Data synthesis	15a	Describe criteria under which study data will be quantitatively synthesised	n/a
	15b	If data are appropriate for quantitative synthesis, describe planned summary measures, methods of handling data and methods of combining data from studies, including any planned exploration of consistency (such as I^2, Kendall's τ)	n/a
	15c	Describe any proposed additional analyses (such as sensitivity or subgroup analyses, meta-regression)	n/a
	15d	If quantitative synthesis is not appropriate, describe the type of summary planned	7-8
Meta-bias(es)	16	Specify any planned assessment of meta-bias(es) (such as publication bias across studies, selective reporting within studies)	n/a

For peer review only

Confidence in cumulative evidence	17	Describe how the strength of the body of evidence will be assessed (such as GRADE)	7

Moher D, Shamseer L, Clarke M, Ghersi D, Liberati A, Petticrew M, Shekelle P, Stewart LA. Preferred Reporting Items for Systematic Review and Meta-Analysis Protocols (PRISMA-P) 2015 statement. Syst Rev. 2015;4(1):1.

Shamseer L, Moher D, Clarke M, Ghersi D, Liberati A, Petticrew M, Shekelle P, Stewart LA, the PRISMA-P Group. Preferred Reporting Items for Systematic Review and Meta-Analysis Protocols (PRISMA-P) 2015: elaboration and explanation. BMJ 2015.349:g7647.

For peer review only

BMJ Open

Food insecurity and the nutritional health and wellbeing of women and children in high-income countries: protocol for a qualitative systematic review

Journal:	*BMJ Open*
Manuscript ID	bmjopen-2020-048180.R1
Article Type:	Protocol
Date Submitted by the Author:	08-Jul-2021

Complete List of Authors:	Bell, Zoe; Newcastle University Faculty of Medical Sciences, Population Health Sciences Institute<br>Scott, Steph; Newcastle University Faculty of Medical Sciences, Population Health Sciences Institute<br>Visram, Shelina; Newcastle University Faculty of Medical Sciences, Population Health Sciences Institute<br>Rankin, Judith; Newcastle University, Population Health Sciences Institute<br>Bambra, Clare; Newcastle University<br>Heslehurst, Nicola; Newcastle University

**Primary Subject Heading**:	Public health
Secondary Subject Heading:	Qualitative research
Keywords:	QUALITATIVE RESEARCH, PUBLIC HEALTH, NUTRITION & DIETETICS

SCHOLARONE™
Manuscripts

I, the Submitting Author has the right to grant and does grant on behalf of all authors of the Work (as defined in the below author licence), an exclusive licence and/or a non-exclusive licence for contributions from authors who are: i) UK Crown employees; ii) where BMJ has agreed a CC-BY licence shall apply, and/or iii) in accordance with the terms applicable for US Federal Government officers or employees acting as part of their official duties; on a worldwide, perpetual, irrevocable, royalty-free basis to BMJ Publishing Group Ltd ("BMJ") its licensees and where the relevant Journal is co-owned by BMJ to the co-owners of the Journal, to publish the Work in this journal and any other BMJ products and to exploit all rights, as set out in our licence.

The Submitting Author accepts and understands that any supply made under these terms is made by BMJ to the Submitting Author unless you are acting as an employee on behalf of your employer or a postgraduate student of an affiliated institution which is paying any applicable article publishing charge ("APC") for Open Access articles. Where the Submitting Author wishes to make the Work available on an Open Access basis (and intends to pay the relevant APC), the terms of reuse of such Open Access shall be governed by a Creative Commons licence – details of these licences and which Creative Commons licence will apply to this Work are set out in our licence referred to above.

Other than as permitted in any relevant BMJ Author's Self Archiving Policies, I confirm this Work has not been accepted for publication elsewhere, is not being considered for publication elsewhere and does not duplicate material already published. I confirm all authors consent to publication of this Work and authorise the granting of this licence.

For peer review only

Food insecurity and the nutritional health and wellbeing of women and children in high-income countries: protocol for a qualitative systematic review

Zoë Bell*[1], Dr Steph Scott[2], Dr Shelina Visram[3], Prof. Judith Rankin[4], Prof. Clare Bambra[5], Dr Nicola Heslehurst [1]

*Corresponding author: Zoë Bell: z.bell2@ncl.ac.uk

[1] nicola.heslehurst@newcastle.ac.uk Population Health Sciences Institute, Newcastle University, Newcastle upon Tyne, NE1 4LP

[2] steph.scott@newcastle.ac.uk Population Health Sciences Institute, Newcastle University, Newcastle upon Tyne, NE1 4LP

[3] shelina.visram@newcastle.ac.uk Population Health Sciences Institute, Newcastle University, Newcastle upon Tyne, NE1 4LP

[4] judith.rankin@newcastle.ac.uk Population Health Sciences Institute, Newcastle University, Newcastle upon Tyne, NE1 4LP

[5] clare.bambra@newcastle.ac.uk Population Health Sciences Institute, Newcastle University, Newcastle upon Tyne, NE1 4LP

Keywords: Food insecurity, Nutrition, Qualitative research, Systematic review

Word count: 3,521 excluding references, title page, figures and tables

For peer review only - http://bmjopen.bmj.com/site/about/guidelines.xhtml

Abstract

Introduction. Since the global financial crises of 2008, there has been a rise in the number of people experiencing food insecurity. The COVID-19 pandemic has exacerbated this. Many more are unable to afford or access food of sufficient quality and quantity to enable good health and wellbeing. Particularly vulnerable are mothers with young children, pregnant women, and lone parents (the majority of whom are women). This review aims to understand experiences of food insecurity and how it affects women and children's nutritional health and wellbeing, focusing on experiences explicitly related to nutrition. Findings will help guide health policy and practice to support food insecure women and children from high-income countries.

Methods and analysis. A systematic review and meta-ethnography exploring i) food insecure women's own accounts of their nutritional health and ii) food insecure household's accounts of their children's nutritional health. Six major databases (Medline, Scopus, Web of Science, EMBASE, CINAHL and ASSIA), grey literature databases and relevant stakeholder websites will be searched from 1st January 2008 to 30th March 2021. Reference list and citation searches will supplement electronic database searches. Outcomes of interest are accounts of nutrition and nutritional health, including diet, food practices, infant feeding practices, physical and mental health. The review will follow the Preferred Reporting Items of Systematic Reviews and Meta-Analyses Protocol (PRISMA) guidelines, but as this is a meta-ethnography will adhere to eMERGe Reporting Guidance for synthesis and writing findings of the final report. Critical Appraisal Skills Programme (CASP) qualitative checklist will assess quality of studies. A meta-ethnographic analysis will be conducted for all included studies.

Ethics and dissemination: As a qualitative systematic review, without primary data collection, ethical approval will not be required. Findings will be submitted for peer-reviewed publication.

Our systematic review protocol was registered with PROSPERO on 23rd October 2020 (registration number CRD42020214159).

Strengths and Limitations of this study

- Employing a rigorous international gold standard methodology (PRISMA-P) to facilitate the development of this protocol and review conduct (PRISMA)
- Adherence to eMERGe Reporting Guidance for synthesising and writing-up the findings of the review
- Comprehensive broad search strategy, supplemented with reference and citation searches
- A potential limitation of the review is that studies will be from different countries with different welfare states, social security, food aid and healthcare systems, which may impact upon temporal relevancy of findings.

For peer review only

Introduction

The terms food insecurity and food poverty both signify “the inability to consume an adequate quality or sufficient quantity of food in socially acceptable ways, or the uncertainty that one will be able to do so” (1). Accessing food in a socially acceptable way means people don’t have to live off ‘free’ food re-distributed by charity (2). In high-income countries (HICs), food insecurity is generally not the result of supply failures but retrenchment of welfare states and diminishing rights to access healthy food (3-6). The financial crises of 2008 meant economic downturn for all HICs, with countries responding differently to the crises. However, since 2008, poverty rates have increased and reports have consistently documented increased use of foodbanks, a proxy measure of food insecurity (7, 8). The COVID-19 pandemic has worsened food insecurity which was already an urgent public health issue (9). Governments imposed, to varying degrees, lockdown measures that increased social isolation and confinement within the home to reduce the reproduction rate of the virus (10). The effects are likely to be unequal across society and are expected to lead to higher poverty rates, having profound effects on economically vulnerable families (11). Families who were teetering on the edge of just managing may be pushed into food insecurity, thereby experiencing hunger, reduced food consumption, and creating an inability to secure food of sufficient quality and quantity to enable good health and participation in society. Particularly vulnerable are families with young children, pregnant women and lone parents, the majority of which are women (12).

It is well established that a healthy balanced diet is an important factor for health. Preconception health is strongly linked not only to pregnancy outcomes, but life course research evidences this period as crucial for health across generations (13), widely recognised is the importance of nutrition during the first 1000 days of life (conception, pregnancy to two years of age) (14). As the diet of a young child is largely determined by the mother, understanding how food insecurity effects women’s nutritional health and wellbeing is important. To date, most reviews exploring the effects of food insecurity on nutrition have reviewed quantitative studies (15-18). Quantitative analyses reveal that individuals experiencing food insecurity are more likely to have a poorer quality diet than those who are food secure (19). Food insecurity is adversely associated with dietary quality for adults; consuming fewer vegetables, fruit, and dairy products than food secure adults and having lower intake of vitamins A, B-6, calcium, magnesium, iron and zinc (16). For children, food insecurity is less consistently associated with lower dietary quality although there is substantial evidence of lower intake of fruit and vegetables (16). Factors influencing the relationship between food insecurity and diet quality remains unclear.

Quantitative studies also report on nutrition-related health outcomes, food insecurity being associated with many non-communicable diseases (15, 20). A major public health concern is the robust association of food insecurity with obesity amongst children and adults in HICs (15, 20-23). Substantial risks for maternal and child health are associated with mothers who are overweight (13). Explanations for the relationship between food insecurity and nutrition-related health remain unclear. Proposed theoretical explanations include sacrifice theory where mothers forego food for their child (supported by the strong relationship between food insecurity and obesity for mothers), substitution hypothesis whereby nutrient-dense, low-energy foods are substituted for cheaper, energy-dense often higher fat and sugar-containing foods (24) and the insurance hypothesis associated with the cyclical nature of food insecurity and its disruption on metabolism (25). Emotional distress has also been theorised as important in the relationship between food insecurity and nutrition-related health (26, 27).

Qualitative research can provide deeper insight into this area. A body of qualitative evidence is accumulating documenting women and children’s experiences and perceptions of food insecurity in relation to their nutritional health and wellbeing. Food insecure participants report altered diets via restrictive eating patterns, for example, reducing portion size, skipping meals or forgoing an entire day of food (28-30). Food insecure participants also report worsened health issues, such as stress, depression and weight gain, which has a knock-on negative effect for the health and wellbeing of dependent children (28, 31) (32). Breastfeeding is the healthiest method for infant feeding, yet a paradox exists where infants from lower-income households are

For peer review only - http://bmjopen.bmj.com/site/about/guidelines.xhtml

least likely to be breastfed, or for as long, despite arguably gaining most from the health and cost benefits it provides (33, 34). Reviewing qualitative research will help to develop an understanding of the complexities around food insecurity and nutritional health and wellbeing.

Furthermore, despite most health research for children and young people being based on parent's, caregivers or stakeholders' views, there is evidence that parents are not always aware of their children's experiences of food insecurity (35). Studies have reported inconsistencies between parent's versus children's perspectives of what the child is experiencing in the household (35). Indeed, evidence shows that children experience food insecurity in different ways to adults (36-38). Systematically reviewing the literature for family unit's perspectives of the effects on food insecurity on children's nutritional health and wellbeing is important to explore the differences and gain a holistic view of it effect.

This review is part of a wider PhD project which explores the social determinants of food insecurity and its nutritional impact amongst women and children, focusing on the first 1000 days of life. The aim of this qualitative systematic review is to explore food insecure women's and children's experiences of nutritional health and wellbeing in HICs, within the context of the last 12 years. This review will further our understanding, inform health policy and practice, and result in recommendations for researchers and areas for further research.

Methods

Preliminary searches were carried out in September 2020 and the study registered with PROSPERO (39) on 23rd October 2020 (registration number CRD42020214159). The review started November 2020 and is anticipated to take eleven months to complete. The review employed a rigorous international gold standard methodology (PRISMA-P) to facilitate the development of this protocol and review conduct (PRISMA) (see supplementary file A).

The review will use meta-ethnography, one of the most developed and structured methods to synthesise qualitative findings, developed by Noblit and Hare (1988) (40). Meta-ethnography involves a seven-phase approach: 1. Getting started 2. Deciding what is relevant to the initial interest 3. Reading the studies 4. Determining how the studies are related 5. Translating the studies into one another 6. Synthesising translations 7. Expressing the synthesis.

Review questions

1. How do women perceive food insecurity to affect their nutrition and nutritional health?
2. How do food insecure household's perceive food insecurity to affect their child's nutrition and nutritional health?

Objectives

The study objectives are:

1. To explore food insecure women's experiences and accounts of their own nutritional health
2. To explore food insecure household's accounts of their children's nutritional health experiences

Eligibility criteria

Studies will be selected according to the inclusion and exclusion criteria outlined in table 1. Eligibility criteria are outlined in accordance with the modified PICO tool, PICOS, which is deemed appropriate for use in qualitative evidence reviews (41, 42).

Table 1 Eligibility Criteria for screening of studies

	Inclusion	Exclusion

For peer review only - http://bmjopen.bmj.com/site/about/guidelines.xhtml

**Participant (P)**	Food insecure women of childbearing age of all ethnicities (objective one)<br><br>Food insecure households (parents, primary caregivers, children) of all ages and ethnicities (objective two)	Studies restricted to a specific type of population not directly related to women and children/ wider population with clinical needs, that necessitates a specific diet (e.g. studies in the context of people living with HIV, type 1 diabetes etc.)<br><br>Studies based on university campus with college students (unless in the context of also being a parent)<br><br>Perspectives of those outside the household family unit i.e. grandparents, healthcare professionals, teachers or food programme coordinators (Objective Two)
**Intervention (I) or Exposure**	Food insecurity.<br><br>Other terms used to describe food insecurity and included are for example food poverty, food deprivation, food insufficiency, hunger.	Food secure population groups<br><br>Studies that were qualitative process evaluations of food insecurity-related interventions/services and focused on women and/or children
**Comparison (C)**	Not applicable – systematic review of qualitative studies	
**Outcomes (O)**	Experiences and accounts of the effect of food insecurity on nutrition and nutritional health and wellbeing	Experiences and accounts not explicitly related to food.
**Study type**	Qualitative studies of any design including but not limited to:<br><br>Ethnography, interviews, focus groups, photo elicitation, visual techniques, phenomenology, grounded theory, case study, feminist research, action research.	Quantitative studies<br><br>Reviews<br><br>Expert opinion articles<br><br>Editorials

For peer review only

	Mixed method studies<br><br>Primary data sources from grey literature and relevant stakeholder websites	Policy documents<br><br>Conference abstracts<br><br>Qualitative research that reports no lay perspectives but has analysed text i.e. discourse analysis<br><br>Grey literature that does not include primary qualitative data
**Study period**	Published in the last 12 years (1 Jan 2008- 2021)<br><br>Studies with data collected from 2008 onwards	Literature published before 1 Jan 2008<br><br>Studies reporting data only collected before 2008
**Setting**	High-income countries (as per OECD definition, see supplementary file B)	Non-high-income countries
**Study reporting language**	English	

For peer review only

Exposure

This review will explore the experiences and accounts of those who are food insecure. Food insecurity is defined as 'the inability to consume an adequate quality or sufficient quantity of food in socially acceptable ways, or the uncertainty that one will be able to do' (1). Other terms used to describe food insecurity are included, for example food poverty, food deprivation, food insufficiency and hunger. In this review, we will include those who are experiencing life on low-income, in receipt of income benefit, accessing food aid and those accessing food through public health programmes for example WIC, SNAP and Healthy Start Vouchers.

Outcomes

Primary

Studies will be included if they report experiences and accounts relating to the effect of food insecurity on nutrition and nutritional health. In the context of this review, nutrition outcomes can be reported as accounts of diet (quality and quantity of food, eating behaviour, eating pattern), food practices (i.e. food acquisition, food preparation, organisation and storage of food in the house) and infant feeding practices (breastfeeding, infant formula and complimentary feeding behaviour). Nutritional health and wellbeing outcomes for women and children include physical (e.g. perspectives on their weight or growth and development of a child) and mental (e.g. anxiety about household food running out).

For peer review only - http://bmjopen.bmj.com/site/about/guidelines.xhtml

Studies exploring food insecure women and children's health more broadly will not be included unless the health effects are explicitly linked to nutrition. Examples of non-nutrition related health effects of food insecurity include stress, anxiety or depression experienced in the context of being unable to pay the household bills or generally living in poverty where there is no mention of nutrition.

Study exclusion

Studies will be excluded if they were published prior to 2008. The year 2008 was selected as a start-date because this marks the beginning of the global financial crises, since which there has been a rise in the number of people experiencing food insecurity. Studies will be excluded if data collection took place prior to 2008. This is to capture the experiences of food insecurity post-global financial crises. Only HICs will be included. See Supplementary File B for a list of HICs as per OECD high-income economies definition (43).

Study design

Qualitative studies of any design will be included if they report experiences and accounts of food insecure women (objective 1) and / or experiences and accounts of household (parents, primary caregivers, children, objective 2) nutrition and nutritional health. Mixed method studies will be included if qualitative data can be extracted independently from quantitative data. Primary data sources from grey literature relevant stakeholder websites will be included if they report primary qualitative data relating to the review.

Search Strategy

We will search six bibliographic databases: MEDLINE, EMBASE, CINAHL, Scopus, Applied Social Science Index and Abstracts (ASSIA) and Web of Science from 1st January 2008 until 30th March 2021. Theses and dissertations will be searched using Open Access Theses and Dissertations (OATD). Grey literature will be searched using OpenGrey Europe (for information on Grey Literature in Europe) and Trove (links to Australian grey literature). Relevant stakeholder websites will be searched in March 2021 (see Supplementary File C). Only publications in English language will be included.

Search terms were identified from literature within the field, and the search strategy has been designed and piloted with an information scientist at Newcastle University. The strategy consists of four main concepts in accordance with the PICOS tool (42) (table 1). An example search strategy for Scopus can be found in Supplementary File D.

The descriptive titles of qualitative studies often lead to inappropriate indexing, posing challenges in finding relevant studies when searching bibliographic databases alone (44). For this reason, we will screen included studies reference lists for other studies published prior to the included study and use citation searching for studies citing the included study, using Google Scholar.

Study Selection

Studies will be imported into EndNote version X9.3.3 (45)for de-duplication, then imported into Rayyan (46) an online program for systematic reviews. All titles and abstracts will be screened by ZB and a second reviewer (split between SS, SV and NH). Full texts will also be double screened. A pilot exercise screening 30 titles and abstracts will be carried out across the screening team (ZB, SS, SV) to calibrate and test the full-text review form. A third reviewer will assist to resolve any disagreements. Reasons for exclusion at the full-text stage will be recorded and a PRISMA flowchart will be used to report each stage of screening.

A standardized data extraction table will be created as a data extraction form and piloted using a sub-set of included studies. The following key information will be extracted from included studies: aim(s), country, time period, population characteristics (e.g. who the accounts and experiences are from i.e. women, pregnant women or breastfeeding, parents, caregivers or children, age ranges, income/employment/SES), sample size, study methods, summary of the main themes with some exemplary direct quotes and context of the research.

For peer review only - http://bmjopen.bmj.com/site/about/guidelines.xhtml

During this stage we will actively look for reporting on each study's context to explore in the analysis. This information will form a table of included studies. Data will be extracted by one reviewer (ZB).

Risk of bias assessment

Quality of included studies will be assessed using the Critical Appraisal Skills Programme (CASP) qualitative checklist (47). The checklist covers the primary focus of the paper, appropriateness of the study design, sample recruitment, methodology, analysis and generalisability. Quality appraisal will be undertaken by one reviewer (ZB), a sample of included studies will be double reviewed for quality appraisal to check agreement. CASP appraisals will be used to inform the data synthesis stage and provide an overview of the quality of included studies for context and inform discussion of the strengths and limitations of existing evidence.

Data synthesis

Methods for review synthesis are to be determined through informed conversations of the nature of the evidence available, the review questions and purpose (48). Meta-ethnography places studies side by side to see how key themes can be translated between studies whilst considering similarities and differences across varied contexts. This interpretive approach moves beyond describing or aggregating findings, instead aiming to 'synthesise understanding' (40). If required, to 'synthesise understanding' we will contact authors of studies to check interpretations of important points (49). The integration of findings of multiple studies will enable the development of deeper insights into the understanding of food insecurity in the context of nutrition, which individual studies alone cannot provide.

Meta-ethnography synthesis will be conducted in seven steps using NVivo 10 software (50). Step one, in-depth reading of included studies by three reviewers (ZB, SS and SV). Step two, creation of study sub-sets and line-by-line coding and extracting of first and second order themes. The third step is to determine how studies are related. We will tabulate first order themes (interpretations) and second order themes (interpretation of interpretations) with grouped studies to create 'meta-themes'. The fourth and fifth steps will involve translating studies by checking first and second order concepts and themes against each other. They may be similar (reciprocal) or refutational. Step six is synthesising the translations to create a third order construct. Step seven is expressing the synthesis through dissemination.

A sample of papers will be duplicate-coded and discussed with the review team. This is to view the data through different perspectives (i.e. a form of investigator triangulation) rather than to check for consistency in coding between reviewers, which is a more positivist approach. We will only code data that explicitly links food insufficiency with weight status i.e., we will not code experiences relating to weight status spoken in the context of wider socioeconomic position. ZB will analyse and synthesise the entire dataset. When synthesising and writing-up the findings of the review, we will adhere to eMERGe Reporting Guidance, recommended to ensure complete and transparent reporting of meta-ethnographies (51).

Patient and Public Involvement

Patients and members of the public were not involved in the design of this protocol. We will consult with local community members for dissemination of the review findings.

Discussion

To the best of our knowledge, this is the first systematic review which aims to synthesize and interpret the findings of qualitative studies of food insecurity in relation to nutritional health, focusing on women and children from HICs. We believe it is important to set the study within the context of the last 12 years as, post-financial crisis, all HICs suffered an economic crash alongside increasing poverty rates. Excluding studies with data collected prior to 2008 ensures that the synthesis is contextualized by the effects of the last financial crises and events since, such as the COVID-19 pandemic.

For peer review only - http://bmjopen.bmj.com/site/about/guidelines.xhtml

We recognize that for HICs outside of the USA and Canada qualitative literature in this area is limited. In response to this we have taken a comprehensive approach to our search strategy in terms of searching stakeholder websites to locate relevant grey literature that might contain primary qualitative data. This is supplemented by screening the reference lists and citations of included studies. To maximize rigor and transparency, this qualitative systematic review was designed following established protocols, and will adhere to recommended and validated methods and reporting guidelines.

A potential limitation of the review is that it will include studies from multiple countries. It may be challenging to draw meaningful conclusions from studies involving different welfare states, social security, food aid and healthcare systems (52, 53). However, the study is set within the context of the global financial crises and not the response to this. Deciding to limit the studies to English language only might exclude some relevant studies, particularly from European countries. However, due to funding and time limitations, translation is beyond the scope of this review.

This synthesis is relevant because it is set within the context of the last financial crises and more recently, a global pandemic that is beginning to result in further economic downturn. These world events have the potential to exacerbate health inequalities. It is important that research focusing on health inequalities is shaped by understandings from people who are experiencing them (54, 55). This review does that by aiming to further our understanding of the experiences of food insecure women and children in the context of nutrition. This review will therefore provide valuable insights for future research and help guide health policy and practice to support food insecure women and children from HICs.

Ethics and Dissemination

The review is using secondary data, so ethical approval is not required. Review findings will be disseminated by publication in theses, peer-reviewed academic journal articles, conferences, policy and practice workshops such as those organized by Fuse: the UK Centre for Translational Research in Public Health (www.fuse.ac.uk) and circulated to the general public and stakeholder groups using social media.

Author Contributions

ZB drafted the manuscript and is the guarantor for the review. All authors (ZB, NH, SS, SV, CB, JR) contributed to the development of the selection criteria. ZB, NH, SS, SV contributed to the quality appraisal assessment strategy and data extraction criteria. ZB developed the search strategy. All authors (ZB, NH, SS, SV, CB, JR) provided comments and amendments. All authors (ZB, NH, SS, SV, CB, JR) read, provided feedback and approved the final manuscript.

Funding Statement

This systematic review was supported by the Economic and Social Research Council (ESRC), as part of a doctoral research project. No award or grant number received. The funder had no role in developing the systematic review protocol and will not be involved in conducting the review.

Conflicts of interest

None declared.

Abbreviations

HIC: High-income countries

OECD: Organisation for Economic Co-Operation and Development

PRISMA: Preferred Reporting Items for Systematic Reviews and Meta-Analyses

PRISMA-P: Preferred Reporting Items for Systematic Reviews and Meta-Analyses (for Protocols)

For peer review only - http://bmjopen.bmj.com/site/about/guidelines.xhtml

PROSPERO: International prospective register of systematic reviews

SNAP: Supplemental Nutrition Assistance Program

WIC: Supplemental Nutrition Assistance Program for Women, Infants and Children

For peer review only

For peer review only - http://bmjopen.bmj.com/site/about/guidelines.xhtml

Bibliography

1. Dowler E. Food and Poverty in Britain: Rights and Responsibilities. Social Policy & Administration. 2002;36(6):698-717.
2. Riches G. Hunger, food security and welfare policies: Issues and debates in First World societies. Proceedings of the Nutrition Society. 1997;56(1A):63-74.
3. Dowler EA, Kneafsey M, Lambie H, Inman A, Collier R. Thinking about 'food security': engaging with UK consumers. Critical Public Health. 2011;21(4):403-16.
4. Dowler EA, O'Connor D. Rights-based approaches to addressing food poverty and food insecurity in Ireland and UK. Social Science & Medicine. 2012;74(1):44-51.
5. Riches G, Silvasti T. Hunger in the Rich World: Food Aid and Right to Food Perspectives. 2014.
6. Lambie-Mumford H. The growth of food banks in Britain and what they mean for social policy. Critical Social Policy. 2018;39(1):3-22.
7. Davis O, Geiger BB. Did Food Insecurity rise across Europe after the 2008 Crisis? An analysis across welfare regimes. Social Policy and Society. 2017;16(3):343-60.
8. Lambie-Mumford H, Green MA. Austerity, welfare reform and the rising use of food banks by children in England and Wales. Area. 2017;49(3):273-9.
9. Loopstra R. Vulnerability to food insecurity since the COVID-19 lockdown The Food Foundation Kings College London; 2020.
10. Bambra C, Riordan R, Ford J, Matthews F. The COVID-19 pandemic and health inequalities. Journal of Epidemiology and Community Health. 2020;74(11):964.
11. International Labour Organisation. The impact of the COVID-19 pandemic on jobs and incomes in G20 economies. 2020.
12. Annesley C, Himmelweit S. The Impact on Women of the Coalition Spending Review 2010. 2010.
13. Stephenson J, Heslehurst N, Hall J, Schoenaker DAJM, Hutchinson J, Cade JE, et al. Before the beginning: nutrition and lifestyle in the preconception period and its importance for future health. The Lancet. 2018;391(10132):1830-41.
14. Barker DJP. The origins of the developmental origins theory. Journal of Internal Medicine. 2007;261(5):412-7.
15. Gundersen C, Ziliak JP. Food Insecurity And Health Outcomes. Health Affairs. 2015;34(11):1830-9.
16. Hanson KL, Connor LM. Food insecurity and dietary quality in US adults and children: a systematic review. Am J Clin Nutr. 2014;100(2):684-92.
17. Myers CA, Mire EF, Katzmarzyk PT. Trends in Adiposity and Food Insecurity Among US Adults. JAMA Network Open. 2020;3(8):e2012767-.
18. de Oliveira KHD, de Almeida GM, Gubert MB, Moura AS, Spaniol AM, Hernandez DC, et al. Household food insecurity and early childhood development: Systematic review and meta-analysis. Matern Child Nutr. 2020;16(3):e12967-e.
19. Yang TC, Sahota P, Pickett KE, Bryant M. Association of food security status with overweight and dietary intake: exploration of White British and Pakistani-origin families in the Born in Bradford cohort. Nutrition Journal. 2018;17(1):48.
20. Larson NI, Story MT. Food insecurity and weight status among U.S. children and families: a review of the literature. Am J Prev Med. 2011;40(2):166-73.
21. Townsend MS, Peerson J, Love B, Achterberg C, Murphy SP. Food Insecurity Is Positively Related to Overweight in Women. The Journal of Nutrition. 2001;131(6):1738-45.
22. Dinour LM, Bergen D, Yeh MC. The food insecurity-obesity paradox: a review of the literature and the role food stamps may play. J Am Diet Assoc. 2007;107(11):1952-61.
23. Franklin B, Jones A, Love D, Puckett S, Macklin J, White-Means S. Exploring mediators of food insecurity and obesity: a review of recent literature. J Community Health. 2012;37(1):253-64.
24. Drewnowski A. Obesity and the food environment: dietary energy density and diet costs. Am J Prev Med. 2004;27(3 Suppl):154-62.
25. Nettle D, Andrews C, Bateson M. Food insecurity as a driver of obesity in humans: The insurance hypothesis. Behav Brain Sci. 2017;40:e105.
26. Hemmingsson E. A new model of the role of psychological and emotional distress in promoting obesity: conceptual review with implications for treatment and prevention. Obesity Reviews. 2014;15(9):769-79.

For peer review only - http://bmjopen.bmj.com/site/about/guidelines.xhtml

27. Hemmingsson E. Early Childhood Obesity Risk Factors: Socioeconomic Adversity, Family Dysfunction, Offspring Distress, and Junk Food Self-Medication. Curr Obes Rep. 2018;7(2):204-9.
28. Harvey K. "When I go to bed hungry and sleep, I'm not hungry": Children and parents' experiences of food insecurity. Appetite. 2016;99:235-44.
29. Puddephatt J-A, Keenan GS, Fielden A, Reaves DL, Halford JCG, Hardman CA. 'Eating to survive': A qualitative analysis of factors influencing food choice and eating behaviour in a food-insecure population. Appetite. 2020;147:104547.
30. Purdam K, Garratt EA, Esmail A. Hungry? Food Insecurity, Social Stigma and Embarrassment in the UK. Sociology. 2015;50(6):1072-88.
31. Garthwaite K, Collins PJ, Bambra C. Food for Thought: An Ethnographic Study of Negotiating Ill Health and Food Insecurity in a UK Foodbank. Social science & medicine (1982). 2015;132:38-44.
32. Thompson C, Smith D, Cummins S. Understanding the health and wellbeing challenges of the food banking system: A qualitative study of food bank users, providers and referrers in London. Social Science & Medicine. 2018;211:95-101.
33. Venu I, van den Heuvel M, Wong JP, Borkhoff CM, Moodie RG, Ford-Jones EL, et al. The breastfeeding paradox: Relevance for household food insecurity. Paediatr Child Health. 2017;22(4):180-3.
34. van den Heuvel M, Birken C. Food insecurity and breastfeeding. Cmaj. 2018;190(11):e310-e1.
35. Fram MS, Frongillo EA, Jones SJ, Williams RC, Burke MP, DeLoach KP, et al. Children are aware of food insecurity and take responsibility for managing food resources. J Nutr. 2011;141(6):1114-9.
36. Attree P. The Social Costs of Child Poverty: A Systematic Review of the Qualitative Evidence. Children & Society. 2006;20(1):54-66.
37. Ridge T. The Everyday Costs of Poverty in Childhood: A Review of Qualitative Research Exploring the Lives and Experiences of Low-Income Children in the UK. Children & Society. 2011;25(1):73-84.
38. Connell CL, Lofton KL, Yadrick K, Rehner TA. Children's experiences of food insecurity can assist in understanding its effect on their well-being. J Nutr. 2005;135(7):1683-90.
39. National Institute for Health Research. International prospective register of systematic reviews 2020 [Available from: https://www.crd.york.ac.uk/prospero/.
40. Noblit G, Hare D. Meta-Ethnography. Thousand Oaks, California1988. Available from: https://methods.sagepub.com/book/meta-ethnography.
41. Methley AM, Campbell S, Chew-Graham C, McNally R, Cheraghi-Sohi S. PICO, PICOS and SPIDER: a comparison study of specificity and sensitivity in three search tools for qualitative systematic reviews. BMC Health Services Research. 2014;14(1):579.
42. Tacconelli E. Systematic reviews: CRD's guidance for undertaking reviews in health care. The Lancet Infectious Diseases. 2010;10(4):226.
43. The World Bank. Doing Business 2020: OECD High-Income Economies Remain Global Benchmarks on Most Doing Business Indicators The World Bank, 2019 [Available from: https://www.worldbank.org/en/news/press-release/2019/10/24/doing-business-2020-oecd-high-income-economies-remain-global-benchmarks-on-most-doing-business-indicators.
44. Atkins S, Lewin S, Smith H, Engel M, Fretheim A, Volmink J. Conducting a meta-ethnography of qualitative literature: Lessons learnt. BMC Medical Research Methodology. 2008;8(1):21.
45. Clarivate Analytics. EndNote X9.3.3 ed.
46. Institutue QCR. Rayyan QCRI. 2020.
47. Singh J. Critical Appraisal Skills Programme. CASP Appraisal Tools. Journal of Pharmacology and Pharmacotherapeutics. 2013;4:76.
48. Booth A. Systematic approaches to a successful literature review. London; Thousand Oaks, California: Sage; 2012.
49. Green J, Thorogood N. Qualitative Methods for Health Research. SAGE. 2004;9:177-80.
50. International Q. NVivo. 11 ed2020.
51. France EF, Uny I, Ring N, Turley RL, Maxwell M, Duncan EAS, et al. A methodological systematic review of meta-ethnography conduct to articulate the complex analytical phases. BMC Medical Research Methodology. 2019;19(1):35.
52. Hillier-Brown F, Thomson K, McGowan V, Cairns J, Eikemo TA, Gil-Gonzále D, et al. The effects of social protection policies on health inequalities: Evidence from systematic reviews. Scandinavian Journal of Public Health. 2019;47(6):655-65.

For peer review only - http://bmjopen.bmj.com/site/about/guidelines.xhtml

53. Hamnett C. Shrinking the welfare state: the structure, geography and impact of British government benefit cuts. Transactions of the Institute of British Geographers. 2014;39(4):490-503.
54. Popay J. What will it take to get the evidential value of lay knowledge recognised? International Journal of Public Health. 2018;63(9):1013-4.
55. Popay J, Williams G, Thomas C, Gatrell T. Theorising Inequalities in Health: The Place of Lay Knowledge. Sociology of Health & Illness. 1998;20(5):619-44.

For peer review only

Supplementary File A: PRIMSA-P 2015 checklist: recommended items to address in a systematic review protocol.

Section and topic	Item No	Checklist item	Page
**ADMINISTRATIVE INFORMATION**			
Title:			
Identification	1a	Identify the report as a protocol of a systematic review	1
Update	1b	If the protocol is for an update of a previous systematic review, identify as such	n/a
Registration	2	If registered, provide the name of the registry (such as PROSPERO) and registration number	4
Authors:			
Contact	3a	Provide name, institutional affiliation, e-mail address of all protocol authors; provide physical mailing address of corresponding author	1
Contributions	3b	Describe contributions of protocol authors and identify the guarantor of the review	9
Amendments	4	If the protocol represents an amendment of a previously completed or published protocol, identify as such and list changes; otherwise, state plan for documenting important protocol amendments	n/a
Support:			
Sources	5a	Indicate sources of financial or other support for the review	9
Sponsor	5b	Provide name for the review funder and/or sponsor	9
Role of sponsor or funder	5c	Describe roles of funder(s), sponsor(s), and/or institution(s), if any, in developing the protocol	9
**INTRODUCTION**			

For peer review only

Rationale	6	Describe the rationale for the review in the context of what is already known	3-4
Objectives	7	Provide an explicit statement of the question(s) the review will address with reference to participants, interventions, comparators, and outcomes (PICO)	4-5
**METHODS**			
Eligibility criteria	8	Specify the study characteristics (such as PICO, study design, setting, time frame) and report characteristics (such as years considered, language, publication status) to be used as criteria for eligibility for the review	5
Information sources	9	Describe all intended information sources (such as electronic databases, contact with study authors, trial registers or other grey literature sources) with planned dates of coverage	7
Search strategy	10	Present draft of search strategy to be used for at least one electronic database, including planned limits, such that it could be repeated	Supplementary File D
Study records:			
Data management	11a	Describe the mechanism(s) that will be used to manage records and data throughout the review	7-8
Selection process	11b	State the process that will be used for selecting studies (such as two independent reviewers) through each phase of the review (that is, screening, eligibility and inclusion in meta-analysis)	7
Data collection process	11c	Describe planned method of extracting data from reports (such as piloting forms, done independently, in duplicate), any processes for	7

For peer review only

		obtaining and confirming data from investigators	
Data items	12	List and define all variables for which data will be sought (such as PICO items, funding sources), any pre-planned data assumptions and simplifications	7
Outcomes and prioritization	13	List and define all outcomes for which data will be sought, including prioritization of main and additional outcomes, with rationale	6
Risk of bias in individual studies	14	Describe anticipated methods for assessing risk of bias of individual studies, including whether this will be done at the outcome or study level, or both; state how this information will be used in data synthesis	7
Data synthesis	15a	Describe criteria under which study data will be quantitatively synthesised	n/a
	15b	If data are appropriate for quantitative synthesis, describe planned summary measures, methods of handling data and methods of combining data from studies, including any planned exploration of consistency (such as I^2, Kendall's τ)	n/a
	15c	Describe any proposed additional analyses (such as sensitivity or subgroup analyses, meta-regression)	n/a
	15d	If quantitative synthesis is not appropriate, describe the type of summary planned	7-8
Meta-bias(es)	16	Specify any planned assessment of meta-bias(es) (such as publication bias across studies, selective reporting within studies)	n/a

For peer review only

Confidence in cumulative evidence	17	Describe how the strength of the body of evidence will be assessed (such as GRADE)	7

Moher D, Shamseer L, Clarke M, Ghersi D, Liberati A, Petticrew M, Shekelle P, Stewart LA. Preferred Reporting Items for Systematic Review and Meta-Analysis Protocols (PRISMA-P) 2015 statement. Syst Rev. 2015;4(1):1.

Shamseer L, Moher D, Clarke M, Ghersi D, Liberati A, Petticrew M, Shekelle P, Stewart LA, the PRISMA-P Group. Preferred Reporting Items for Systematic Review and Meta-Analysis Protocols (PRISMA-P) 2015: elaboration and explanation. BMJ 2015.349:g7647.

For peer review only

Supplementary File B: List of high-income countries that meet the inclusion criteria (defined by list of OECD High-Income Economies). Available at: https://www.worldbank.org/en/news/press-release/2019/10/24/doing-business-2020-oecd-high-income-economies-remain-global-benchmarks-on-most-doing-business-indicators. Accessed November, 2020)

Australia
Austria
Belgium
Canada
Chile
Czech Republic
Denmark
Estonia
Finland
France
Germany
Greece
Hungary
Iceland
Ireland
Israel
Italy
Japan
Korea
Latvia
Lithuania
Luxembourg
Netherlands
New Zealand
Norway
Poland
Portugal
Slovakia
Slovenia
Spain
Sweden
Switzerland
United Kingdom
United States

For peer review only

Supplementary File C - List of relevant stakeholder websites to be searched

- European Food banks Federation - https://www.eurofoodbank.org/
- Trussell Trust - https://www.trusselltrust.org/
- Independent Food Aid Network - https://www.foodaidnetwork.org.uk/
- The Food Foundation - https://foodfoundation.org.uk/
- Feeding Britain - https://feedingbritain.org/
- Feeding America - https://www.feedingamerica.org/
- Foodbanks Canada - https://www.foodbankscanada.ca/
- Foodbank Australia - https://www.foodbank.org.au/?state=au
- The Foodbank Project - https://www.foodbank.org.nz/
- Royal College of Gynaecology and Obstetrics - https://www.rcog.org.uk/
- Royal College of Nursing - https://www.rcn.org.uk/
- World Health Organiaation and affiliate websites (e.g., UNICEF, International Confederation of Midwives) - https://www.who.int/ https://www.unicef.org.uk/ https://www.internationalmidwives.org/
- Paediatrics; Nursing sub-specialties – Perinatal, Neonatal, Community, Family, Public Health; Midwifery, Dietitian and Doula coalitions and associations – websites from high resourced, Western countries (e.g., International Coalition of Nurses https://www.icn.ch/)
- Baby Friendly Initiative (formerly Baby Friendly Hospital Initiative) websites associated with high resourced, Western countries e.g. https://www.unicef.org.uk/babyfriendly/
- Breastfeeding and Lactation coalitions and associations – websites from high resourced, Western countries (e.g., La Leche League International https://www.llli.org/)

For peer review only

Supplementary File D – Example search strategy

**Search Concept – Scopus**			**Rationale**
Population terms	S1	TITLE-ABS-KEY(wom?n OR female OR mother* OR matern* OR mum OR father OR dad* OR caregiver OR guardian OR parent* OR preg* OR postnatal OR newborn OR baby OR babies OR infan* OR toddler OR child* OR preschool OR adolescent OR teenager OR "young adult" OR "lowest income group")	Synonyms for population group from scoping search [1,2,3,4]<br><br>Lowest income group and young adult added as terms to reflect key words of two scoping papers [3,4]<br><br>Quotation marks added so that the database recognises it is a phrase and not two separate words, reducing the number of results
Intervention terms	S2	TITLE-ABS-KEY("food insecur*" or "food secur*" or "food poverty" or "food insufficien*" or "food assistance" or "food depriv*" or "poverty" or "foodbanks" or "food banks" or "hunger")	Key terms/synonyms from scoping search [2-6]
Outcome terms	S3	TITLE("nutrition" or "food practices" or "food preferences" or "healthy eating" or "feeding behaviour" or "feeding practices" or "food habits" or "diet" or "diet quality" or "portion size" or "breastfeeding" or "complimentary feeding" or "weaning" or "maternal nutrition" or "child nutrition" or "infant food")	Key terms/synonyms of outcomes of this review [3-7]
	S4	TITLE("weight" or "obesity" or "childhood obesity" or "development" or "growth")	Key terms for nutrition-related health outcomes of this review
	S5	S3 or S4	
Study Design terms	S6	"qualitative research" or "grounded theory" or "ethnograph*" or "phenomenolog*" or "feminis*" or "narrative*" or "interview*" or "focus group*" or "case stud*" or "anthrop*" or "observ*" or "field notes" or "biograph*" or "life history" or "photovoice" or "photo elicitation" or "autoethnograph*" or "creative method" or "thematic analysis"	Refine results by study design as this is a qualitative systematic review
	S7	S1 and S2 and S5 and S6	

For peer review only

		(LIMIT-TO (PUBYEAR,2021) OR LIMIT-TO (PUBYEAR,2020) OR LIMIT-TO (PUBYEAR,2019) OR LIMIT-TO (PUBYEAR,2018) OR LIMIT-TO (PUBYEAR,2017) OR LIMIT-TO (PUBYEAR,2016) OR LIMIT-TO (PUBYEAR,2015) OR LIMIT-TO (PUBYEAR,2014) OR LIMIT-TO (PUBYEAR,2013) OR LIMIT-TO (PUBYEAR,2012) OR LIMIT-TO (PUBYEAR,2011) OR LIMIT-TO (PUBYEAR,2010) OR LIMIT-TO (PUBYEAR,2009) OR LIMIT-TO (PUBYEAR,2008)	Further refined the results on the basis that this protocol is set within the context of the last 12 years

For peer review only

		(LIMIT-TO (AFFILCOUNTRY , "United States") OR LIMIT-TO (AFFILCOUNTRY , "United Kingdom") OR LIMIT-TO (AFFILCOUNTRY , "Canada") OR LIMIT-TO (AFFILCOUNTRY , "Australia") OR LIMIT-TO (AFFILCOUNTRY , "Germany") OR LIMIT-TO (AFFILCOUNTRY , "France") OR LIMIT-TO (AFFILCOUNTRY , "Italy") OR LIMIT-TO (AFFILCOUNTRY , "Netherlands") OR LIMIT-TO (AFFILCOUNTRY , "Switzerland") OR LIMIT-TO (AFFILCOUNTRY , "Spain") OR LIMIT-TO (AFFILCOUNTRY , "Sweden") OR LIMIT-TO (AFFILCOUNTRY , "Norway") OR LIMIT-TO (AFFILCOUNTRY , "New Zealand") OR LIMIT-TO (AFFILCOUNTRY , "Japan") OR LIMIT-TO (AFFILCOUNTRY , "Belgium") OR LIMIT-TO (AFFILCOUNTRY , "Denmark") OR LIMIT-TO (AFFILCOUNTRY , "Finland") OR LIMIT-TO (AFFILCOUNTRY , "Poland") OR LIMIT-TO (AFFILCOUNTRY , "Chile") OR LIMIT-TO (AFFILCOUNTRY , "Austria") OR LIMIT-TO (AFFILCOUNTRY , "Ireland") OR LIMIT-TO (AFFILCOUNTRY , "Israel") OR LIMIT-TO (AFFILCOUNTRY , "Portugal") OR LIMIT-TO (AFFILCOUNTRY , "Czech Republic") OR LIMIT-TO (AFFILCOUNTRY , "Greece") OR LIMIT-TO (AFFILCOUNTRY , "Hungary") OR LIMIT-TO (AFFILCOUNTRY , "Lithuania") OR LIMIT-TO (AFFILCOUNTRY , "Luxembourg") OR LIMIT-TO (AFFILCOUNTRY , "Slovakia") OR LIMIT-TO (AFFILCOUNTRY , "Estonia") OR LIMIT-TO (AFFILCOUNTRY , "Iceland") OR LIMIT-TO (AFFILCOUNTRY , "Slovenia") OR LIMIT-TO (AFFILCOUNTRY , "Latvia") OR LIMIT-TO (AFFILCOUNTRY , "Undefined")	Refined results to high income countries as defined by the OECD members list[8]<br><br>This was not added as a concept into the search strategy because of the large number of alternative words associated with a country that could be in the title or abstract. For instance, England / English (Engl*) or Holland / Dutch.<br><br>Another reason was because in the UK, for instance, the existing qualitative evidence base is limited to cities and regions, so country name might not be in the title [9]<br><br>Scoping showed that limiting the search by affiliate country did not exclude key papers
		(LIMIT-TO (LANGUAGE , "English")	Limited to English language

For peer review only

Limit justification:

This search was limited to the last 12 years to set the review because because post financial crises all high-income countries suffered an economic crash, alongside increasing poverty rates and food insecurity

The search was further refined to English language or existing translation in English due to project time constraints and budget. It was limited to human participants given that this is the population of interest.

1 Attree, P. (2004), Growing up in disadvantage: a systematic review of the qualitative evidence. Child: Care, Health and Development, 30: 679-689.

2 Attree, P. (2005), Low-income mothers, nutrition and health: a systematic review of qualitative evidence. Maternal & Child Nutrition, 1: 227-240.

3 Gross RS, Mendelsohn AL, Arana MM, Messito MJ. (2019) Food Insecurity During Pregnancy and Breastfeeding by Low-Income Hispanic Mothers. Pediatrics, 143(6)

4 Harden, J. and Dickson, A. (2015) 'Low-income mothers' food practices with young children: A qualitative longitudinal study', Health Education Journal, 74(4), pp. 381–391

5 Frank, L. (2015) Exploring Infant Feeding Practices In Food Insecure Households: What Is The Real Issue?, Food and Foodways, 23:3, 186-209

6 Lovelace S, Rabiee-Khan, F. (2015) Food choices made by low-income households when feeding their pre-school children: a qualitative study, Maternal Child Nutrition, 11(4), pp. 870-81

7 Zorbas C, Palermo C, Chung A, Iguacel I, Peeters A, Bennett R, Backholer K. (2018) Factors perceived to influence healthy eating: a systematic review and meta-ethnographic synthesis of the literature, Nutrition Reviews, 76(12) pp. 861–874

8 OECD. List of OECD High-Income Economies. Available at: https://www.worldbank.org/en/news/press-release/2019/10/24/doing-business-2020-oecd-high-income-economies-remain-global-benchmarks-on-most-doing-business-indicators. Accessed November, 2020.

9 Puddephatt J-A, Keenan GS, Fielden A, Reaves DL, Halford JCG, Hardman CA. (2020) 'Eating to survive': A qualitative analysis of factors influencing food choice and eating behaviour in a food-insecure population. Appetite, 147:104547.

For peer review only
